# The application of phenylboronic acid pinacol ester functionalized ROS-responsive multifunctional nanoparticles in the treatment of Periodontitis

**DOI:** 10.1186/s12951-024-02461-0

**Published:** 2024-04-15

**Authors:** Jinhong Chen, Aihua Luo, Mengmeng Xu, Yao Zhang, Zheng Wang, Shuang Yu, Li Zhu, Wei Wu, Deqin Yang

**Affiliations:** 1 Department of Endodontics, Stomatological Hospital of Chongqing Medical University, Chongqing, 404100 China; 2 Key Laboratory of Biorheological Science and Technology, College of Bioengineering, Ministry of Education, Chongqing University, Chongqing, 400044 China

**Keywords:** Phenylboronic acid pinacol ester, Hyaluronic acid, ROS-responsive, Curcumin, Periodontitis

## Abstract

Periodontitis is an inflammatory disease induced by the complex interactions between the host immune system and the microbiota of dental plaque. Oxidative stress and the inflammatory microenvironment resulting from periodontitis are among the primary factors contributing to the progression of the disease. Additionally, the presence of dental plaque microbiota plays a significant role in affecting the condition. Consequently, treatment strategies for periodontitis should be multi-faceted. In this study, a reactive oxygen species (ROS)-responsive drug delivery system was developed by structurally modifying hyaluronic acid (HA) with phenylboronic acid pinacol ester (PBAP). Curcumin (CUR) was encapsulated in this drug delivery system to form curcumin-loaded nanoparticles (HA@CUR NPs). The release results indicate that CUR can be rapidly released in a ROS environment to reach the concentration required for treatment. In terms of uptake, HA can effectively enhance cellular uptake of NPs because it specifically recognizes CD44 expressed by normal cells. Moreover, HA@CUR NPs not only retained the antimicrobial efficacy of CUR, but also exhibited more pronounced anti-inflammatory and anti-oxidative stress functions both in vivo and in vitro. This provides a good potential drug delivery system for the treatment of periodontitis, and could offer valuable insights for dental therapeutics targeting periodontal diseases.

**Supplementary Information:**

The online version contains supplementary material available at 10.1186/s12951-024-02461-0.

## Introduction

Periodontitis, as an inflammatory disease that inflicts damage on the supportive tissues of teeth, leads to the destruction of both soft and hard tissues, e.g. the gingiva, periodontal ligament, cementum, and alveolar bone [1]. Plaque microorganisms and their metabolites are the initiating factors in the development of periodontitis, while the host immune response plays a pivotal role in the destruction of periodontal tissue. Periodontal tissues release excessive amounts of inflammatory mediators and reactive oxygen species (ROS) due to continuous stimulation by pathogenic microorganisms. These will lead to the destruction of periodontal hard and soft structures. Currently, conventional treatments for periodontitis have certain limitations, such as difficulties in eliminating bacteria and calculus residing deep within periodontal pockets. Therefore, the effectiveness of current therapeutic approaches in modulating inflammation and clearing ROS is considerably limited [2]. In addition, adjuvant drugs such as antibiotics, anti-inflammatory drugs and probiotics, they can overcome some of the limitations of mechanical therapy to a certain extent [3]. However, due to their single mode of action, they are unable to remove overexpressed ROS during the inflammatory process [4]. Under these circumstances, it is envisaged that effective local anti-inflammatory and ROS scavenging drugs or drug carriers may modify the periodontal microenvironment and attenuate inflammation as well as the pathological process of high levels of ROS. It has been widely demonstrated that many natural products possess antimicrobial, antioxidant, and anti-inflammatory properties and have been extensively utilized in treating various diseases including cancer, malaria, and periodontitis [5].

Curcumin (CUR), as a polyphenolic compound, has antimicrobial, antioxidant and anti-inflammatory activities. It also has the advantages of low toxicity, few adverse effects and low price [6, 7]. Pulikkotil et al [8]. divided 60 volunteers with healthy gingiva into three groups: a CUR gel group, a chlorhexidine gel group, and a metronidazole (MTZ) gel group, with applications twice daily for 29 days. The results indicated that the anti-inflammatory effectiveness of CUR gel was comparable to that of MTZ gel and superior to chlorhexidine gel. However, CUR still suffers from deficiencies such as low water solubility, low bioavailability, unstable structure, rapid metabolism, and inadequate dispersion, which limit its clinical applications. It is well known that nano-delivery systems have the advantages of enhancing drug solubility, increasing drug stability, reducing toxicity and decreasing drug degradation [9–11]. This provides a more advanced method of drug delivery for anti-periodontitis. The smaller size of nano-delivery systems allows them to reach sites that are inaccessible to other instruments, such as deep periodontal pockets [12]. For example, Zambrano et al [13] prepared biocompatible periodontitis nanoparticles by encapsulating curcumin nanoparticles with poly(lactic acid)-poly(hydroxybutyacid) copolymer (PLGA). The results showed that topical application of curcumin nanoparticles could inhibit alveolar bone resorption, reduce the number of osteoclasts, and attenuate the inflammatory response, which was a good therapeutic effect for periodontitis. However, these nano-delivery systems still face many limitations. The scouring effect of saliva in the mouth makes it difficult for the drug to stay in the periodontal tissue for a long time, which reduces the therapeutic effect of the drug. Therefore, the design of a nanocarrier that can be retained in the local microenvironment as well as specifically released is important for the treatment of periodontitis.

Hyaluronic acid (HA), a natural linear polysaccharide composed of alternating units of N-acetylglucosamine and D-glucuronic acid, is a principal component of the extracellular matrix and cellular interstitial material [12, 14]. HA is capable of specific binding with various receptors, such as CD44. These receptors play multiple roles in cellular functions, including participating in inflammatory responses and endocytosis [13]. Based on the high expression of CD44 on macrophages, fibroblasts and epithelial cells, a receptor-ligand interaction strategy was used to design an actively targeted periodontal drug delivery system to promote uptake. Furthermore, under the action of lysosomal enzymes, HA can be degraded into N-acetylglucosamine and glucuronic acid. These are subsequently metabolized by the body and excreted as carbon dioxide, water, and urea. Hence, HA possesses excellent biocompatibility and biodegradability. Additionally, ROS-responsive biomaterials have been widely used for tissue regeneration and the treatment of inflammatory diseases such as cardiovascular disease, osteoarthritis, chronic diabetic wounds, and inflammatory bowel disease [15]. However, ROS-responsive drug delivery systems have been less studied in the treatment of periodontitis. Excess ROS generated during periodontitis pathology can directly damage periodontal tissues through DNA damage and protein denaturation, and can also aggravate periodontitis by promoting the production and expression of pro-inflammatory cytokines and chemokines [16, 17]. Therefore, designing a ROS-responsive drug delivery system to achieve specific and controlled release of drugs by altering ROS levels in the pathological microenvironment of periodontitis will effectively improve the therapeutic efficiency of periodontitis.

**Scheme 1:**
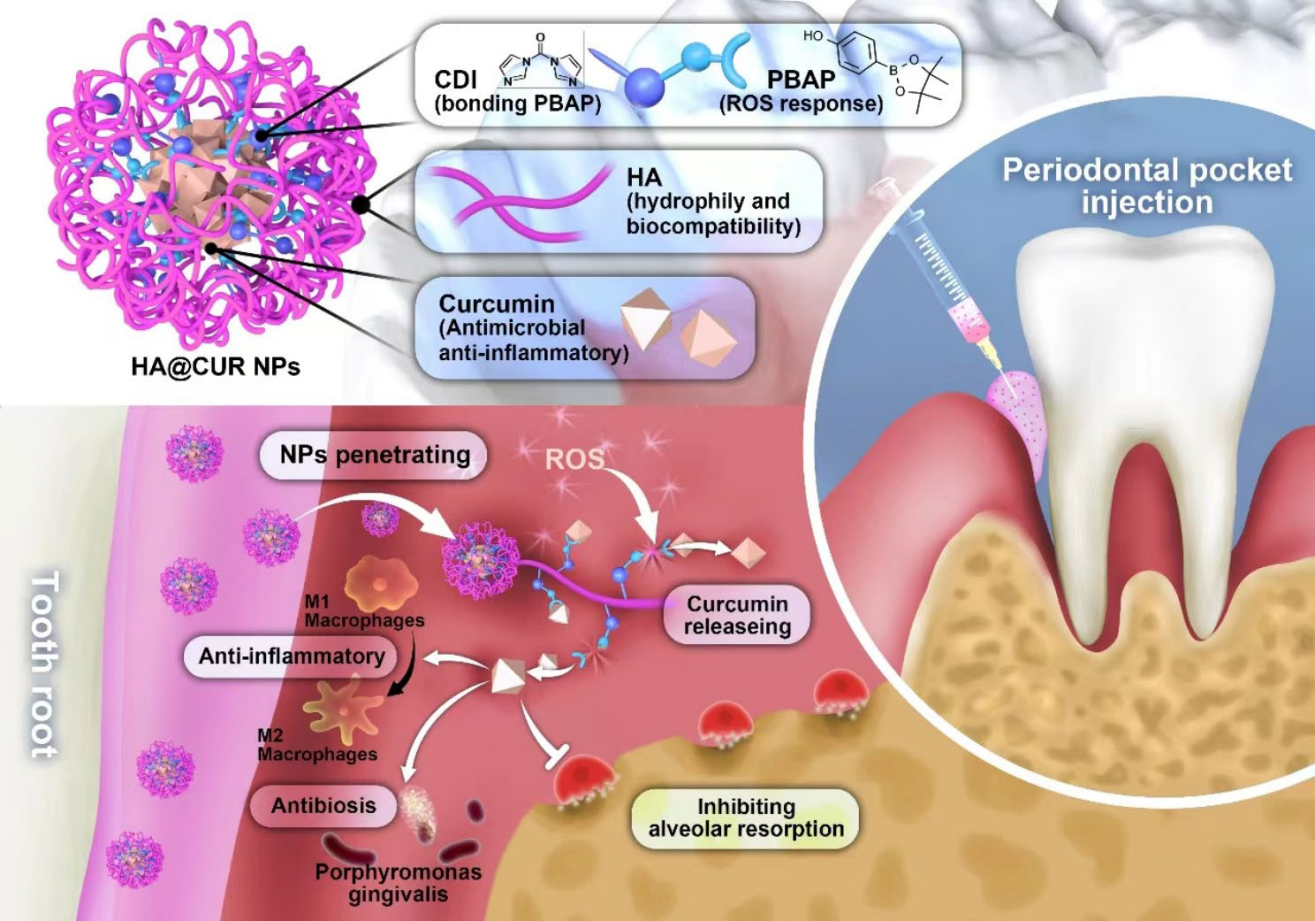
The application of HA@CUR NPs as ROS-responsive multifunctional nanoparticles in the treatment of periodontitis, and an explanation of their potential mechanism for alleviating chronic inflammation in an SD rat model

Based on above discussion, we structurally modified HA using pinacol ester of 4-hydroxybenzeneboronic acid (PBAP) to prepare HA-PBAP ROS-responsive smart nano-loading system, which was able to encapsulate CUR to form CUR NPs (HA@CUR NPs) (Scheme 1). This system uses the hydrophilic HA-PBAP to encapsulate CUR, which possesses a diverse array of biological functions including antibacterial, antioxidant, anti-inflammatory, and immunomodulatory effects. PBAP has a sensitive ROS responsiveness. In the presence of ROS, the CUR inside HA@CUR NPs is quickly released and ensures the structural integrity of the drug in the process. Compared with non-responsive NPs, the ROS-responsive delivery system was able to specifically release the drug and increase the drug concentration at the site of inflammation. In vitro results showed that HA@CUR NPs inhibited and killed *Porphyromonas gingivalis (P.g.)*. Moreover, HA@CUR NPs had the functions of scavenging excessive ROS, down-regulating inflammatory factor levels, and inducing M2-type macrophage production. In addition, these NPs showed good ability to reduce the degree of inflammation, inhibit the number of osteoclasts, and reduce the loss of alveolar bone in an experimental periodontitis model in rats. This study suggests that HA@CUR NPs provide an effective strategy for the treatment of periodontitis.

## Materials and methods

### Reagents and materials

CUR (> 98.0% purity) was obtained from Solarbio (China). 4-Hydroxyphenylboronic acid pinacol ester (PBAP), N,N’-carbonyldiimidazole (CDI), and sodium hyaluronate (HA) were acquired from Macklin (China). The reverse transcription kit was purchased from Accurate Biology (Hunan, China). The RNAeasy™ Animal RNA Extraction Kit and Reactive Oxygen Species Assay Kit were provided by Beyotime (China). The SYBR Prime qPCR Set and Cell Counting Kit-8 (CCK-8) were procured from Bioground (China). Hematoxylin and eosin (H&E) staining kits and tartrate-resistant acid phosphatase staining kits (TRAP Stain Kit) were purchased from Solarbio (China). Cyanine 5.5 (Cy5.5) and lipopolysaccharide (LPS) were sourced from Sigma-Aldrich (USA). Live/Dead BacLight Bacterial Viability Kits were provided by Bestbio (China). All other reagents used were of analytical grade.

### Preparation of PBAP-modified HA (HA-CDI-PBAP)

The preparation of HA-PBAP was carried out with reference to previous reports and improved [18]. First, 4 mM of PBAP and 8 mM of CDI were dissolved in 5 mL of dichloromethane, respectively. These solutions were then combined in a 50 mL round-bottom flask and stirred at 40 °C in an oil bath for 1 hour. The reaction product was washed three times with ultrapure water, followed by an extraction with saline on the fourth wash. Subsequent rotary evaporation yielded pure PBAP-CDI. 6 mM of PBAP-CDI and 1 mM of HA were added to 20 mL of DMSO, and then 7.2 mM of DMAP was added, and ultrasonication was performed to dissolve the reaction, and the reaction was stirred overnight at 40 ℃ in an oil bath, and then dialyzed and lyophilized to obtain the HA-CDI-PBAP. The chemical structure of the material was characterized by Fourier-transform infrared spectroscopy (FT-IR) and proton nuclear magnetic resonance (^1^H-NMR).

### Preparation of CUR nanoparticles (HA@CUR NPs)

CUR (6 mg) was dissolved in 2 mL of dimethyl sulfoxide (DMSO) and mixed with 60 mg of HA-CDI-PBAP under sonication for a co-dissolution process. The resulting solution was then transferred into a dialysis bag (molecular weight cutoff: 3500 kDa) for dialysis. During dialysis, the aqueous solution outside the dialysis bag was replaced every 2 h for a total duration of 6 h. Subsequently, the HA@CUR NPs were obtained through freeze-drying and stored at -20 °C in the dark. Note: Synthesis of HA-Cy5.5 NPs: 100 mg of PBAP-CDI-HA and 251 mg of 1-[3-dimethylaminopropyl]-3-ethylcarbodiimide (EDC) and 151 mg of N-hydroxysuccinimide (NHS) were activated for 8 h in 10 mL DMSO at room temperature. 5 mg Cy5.5-NH_2_ was dissolved in the above solution, supplemented with DMSO as appropriate, reacted for a further 24 h, dialyzed and lyophilized. 5 mg Cy5.5-NH_2_ was dissolved in the above solution, supplemented with DMSO as appropriate, reacted for a further 24 h, then HA-Cy5.5 nanoparticles can be obtained after dialysis and lyophilization.

A UV-visible spectroscopy scan was employed to establish a standard curve of different concentrations of CUR. This curve was then used to determine the encapsulation efficiency and drug loading content of the NPs.

Encapsulation efficiency of CUR (%) = Mass of CUR in NPs / Initial mass of CUR used × 100%.

Drug loading content of CUR (%) = Mass of CUR in NPs / Total mass of NPs × 100%.

### Characterization of HA@CUR NPs

Dynamic light scattering (DLS) measurements of the size distribution and zeta potential of HA@CUR NPs in aqueous solution were conducted on a Nano ZS90 instrument. The stability of HA@CUR NPs was monitored by measuring the size and polydispersity index (PDI) of the nanoparticle solutions stored at 37 ℃ at fixed time intervals.

The morphology and size of the HA@CUR NPs were examined using transmission electron microscopy (TEM).

The interactions between the drug and carrier materials within the NPs were further analyzed by FTIR.

X-ray diffraction (XRD) patterns of free CUR, blank NPs, and HA@CUR NPs were acquired using an X-ray diffractometer (PANalytical X’Pert Powder, Panalytical B.V., Netherlands) operating with Cu Kα radiation. The scattering angles ranged from 3 to 50 degrees.

### Study of ROS responsiveness

#### In vitro ROS-Responsive drug release assay

The ROS-responsive release of the drug from NPs was evaluated using a dialysis method. To begin, 1 mL of HA@CUR NPs solution (concentration: 650 µg/mL) was placed into a dialysis bag with a molecular weight cutoff of 3500 Da. This bag was then submerged in 30 mL of phosphate-buffered saline (PBS) at different H_2_O_2_ concentration (0, 10, 100, 1000 µM) at a pH level of 7.4, and the release was performed at 37 °C with gentle agitation in an incubator. At specific time intervals (0.5, 1, 2, 4, 8, 12, 24, and 48 h), a 3.0 mL aliquot of the dialysate was withdrawn from the exterior of the bag and immediately replenished with an equal volume of fresh release medium to maintain a constant volume. The absorbance was determined using a UV-visible spectrophotometer. Using a standard curve, the concentration of CUR present in the samples was calculated, which allowed for the quantification of the released drug. The cumulative percentage release of CUR was computed as reported previously [14, 19] and presented as the mean ± standard deviation (SD). The cumulative release curve was plotted based on these measurements for graphical representation.

#### ROS-responsive morphological changes

The ROS-responsive process was determined by observing the morphological changes of HA@CUR NPs under different conditions using TEM.

### Cytotoxicity studies

RAW 264.7 cells were seeded at a density of 2,000 cells per well in a 96-well plate, to which 100 µL of Dulbecco’s Modified Eagle’s Medium (DMEM) complete culture medium was added. The plate was then incubated overnight at 37 °C with 5% CO_2_. After 12 h, the medium was replaced with 100 µL of medium containing different concentrations of HA@CUR NPs or CUR, and incubated for 24 h in a 37 ℃, 5% CO_2_ incubator. Subsequently, the original culture medium was discarded and the cells were rinsed thrice with Phosphate-Buffered Saline (PBS), then 100 µL of fresh serum-free medium along with 10 µL of Cell Counting Kit-8 (CCK8) solution were added to each well. The plate was incubated for an additional 3 h at 37 °C in 5% CO_2_. Finally, the absorbance was measured at 450 nm with a multifunctional enzyme marker.

$$\eqalign{{\rm{Cell}}\,{\rm{viability}}\,\left( {\rm{\% }} \right)\,{\rm{ = }}\, & \cr & \,{\rm{[(OD}}\,{\rm{experiment}}\,{\rm{ - }}\,{\rm{OD}}\,{\rm{blank)}} \cr & {\rm{/}}\,{\rm{(OD}}\,{\rm{control}}\,{\rm{ - OD)]}}\, \times \,100\% \cr}$$

Here, OD_experiment represents the optical density values post-treatment with either different concentrations of HA@CUR NPs or CUR. OD_blank denotes the optical density of wells containing only the CCK8 reagent without cells, serving as the blank control. OD_control refers to the optical density of untreated cell wells with no added therapeutic agents.

### Hemolysis assay of nanoparticles

Blood was collected from the hearts of SD rats and stored in vacuum blood collection tubes at 4 °C. Fresh blood was diluted with 0.9% NaCl solution at a volume ratio of 4:5, followed by a water bath at 37 °C for 30 min. HA@CUR NPs and CUR (each group was set at 1.25, 2.5, 5, 10 and 20 µg/mL) were mixed with diluted blood (at a ratio of 1 ml:20 µL) and incubated in a 37 °C water bath for 1 h. After incubation, the mixture was centrifuged at room temperature at 3000 rpm for 10 min, and then photographs were taken to record the result. The supernatant was collected and transferred to a 96-well plate to measure the optical density (OD) at 545 nm. Normal saline (0.9% NaCl) and deionized water served as negative and positive controls, respectively, for calculating the hemolysis percentage.

The hemolysis rate (%) was calculated as follows:

$$\eqalign{{\rm{Hemolysis}}\,{\rm{rate}}\,\left( {\rm{\% }} \right)\,{\rm{ = }}\, & \cr & \left( {{\rm{OD\_experiment - OD\_negative}}} \right){\rm{ }} \cr & {\rm{/}}\,\left( {{\rm{OD\_positive - OD\_negative}}} \right)\,{\rm{ \times }}\,{\rm{100 \% }} \cr}$$

If the hemolysis rate exceeded 5%, the material was deemed to exhibit hemolytic effects.

### *Invitro* cell affinity assay

According previous report [20], RAW 264.7, HGFs, and Caco2 cells were inoculated in 6-well plates at a density of 1 × 10^5^ cells per well. After 12 h of incubation, cells were collected and stained with PE-labelled anti-CD44 (103,007, Biolegend), and the expression of CD44 receptor in the three cells was detected using flow cytometry. The cells were then cultured at a density of 1 × 10^5^ cells per well in 6-well plates for HA targeting evaluation. 10 µg HA-Cy5.5 NPs, 10 µg HA-Cy5.5 NPs + 20 µg HA, 10 µg HA-Cy5.5 NPs + 5 µL CD44 antibody were added to the six-well plates, respectively, and incubated for 2 h. Cells were washed three times and collected for flow cytometry analysis. For comparison, non-activated RAW 264.7 cells were used as a control, and then 10 µg HA-Cy5.5 NPs, 10 µg HA-Cy5.5 NPs + 20 µg HA, 10 HA-Cy5.5 NPs + 5 µL CD44 antibody were added to 6-well plates, incubated for 2 h, and then the cells were washed three times and collected for FCM analysis.

### In vitro cellular uptake

The uptake of HA-Cy5.5 NPs (prepared as described previously) was observed by fluorescence microscopy to verify the targeting of HA. RAW 264.7 cells were inoculated in 6-well plates at a density of 10^4^ cells/well, and 2 mL of DMEM complete medium was added to each well and incubated overnight at 37 ℃ in a 5% CO_2_ incubator. After 12 h, the original medium was aspirated, 10 µg HA-Cy5.5 NPs, 10 µg HA-Cy5.5 NPs + 20 µg HA, 10 µg HA-Cy5.5 NPs + 5 µL CD44 antibody were added to each well of the medium, incubated for 2 h, then the dishes were removed, washed with PBS for 3 times, and 100 µL of Hoechst was added, and the nuclei were static stained for 10 min at room temperature, and cell uptake was observed under a fluorescence microscope to observe the uptake of cells.

### Investigation of nanoparticle antibacterial activity

#### Determination of minimum inhibitory concentration (MIC)

The antibacterial activity of NPs against *P.g.* was assessed using the broth microdilution method [21]. Bacterial suspensions were diluted to a concentration of 10^6^ CFU/mL. Each well of a 96-well plate received 100 µL of the bacterial suspension. The positive control wells contained only bacterial suspension, while the negative control wells contained only the medium. Solutions of CUR and HA@CUR NPs at various concentrations were prepared, sterilized by filtration, and added to the 96-well plate, followed by incubation in a 37 °C anaerobic chamber. After 24 h, the optical density at 600 nm (OD 600) was measured for each well. The antibacterial rate was calculated for different concentrations of CUR and HA@CUR NPs, with the lowest drug concentration achieving a 90% inhibition rate considered the MIC.

#### Antibacterial plate spreading experiment

An appropriate volume of bacterial suspension was taken from each well described above, diluted, and 50 µL of the suspension was inoculated onto blood agar plates. After incubation at 37 °C for 72 h, each group of blood plates was photographed to observe the growth and number of bacterial colonies.

#### Bacterial live/dead staining

Initially, glass coverslips measuring 10 mm were sterilized and placed into 48-well plates. Subsequently, 300 µL of *P.g.* suspension (approximately 10^6^ CFU/mL) was added, followed by incubation at 37 °C in an anaerobic chamber for 24 h to allow biofilm formation. Post incubation, solutions of CUR and HA@CUR NPs at different concentrations were applied, and the culture was further incubated for 12 h. The bacteria were then washed 3 times with PBS. For staining, 100–200 µL of live bacteria stain (N01 solution) and dead bacteria stain (PI solution) were used to incubate the bacteria for 15 min at room temperature in the dark. After staining, the bacteria were washed 3 times with PBS. The coverslips were then placed on microscope slides, and fluorescence microscopy was used for examination and photography.

#### Observation of bacterial surface morphology

A volume of 500 µL containing a 1 × 10^6^ CFU/mL *P.g.* suspension was added to each well of a 24-well plate. The plates were incubated at 37 °C in an anaerobic chamber for 24 h; subsequently, different concentrations of CUR and HA@CUR NPs solutions were added, and the incubation continued for another 12 h. Afterwards, the bacteria were washed 3 times with PBS and then fixed using 2.5% glutaraldehyde solution at 4 °C overnight. Subsequently, after gradient alcohol dehydration treatment, the bacteria were resuspended with tert-butanol, 10 µL of bacterial solution was taken on a drop of conductive gel, dried and sprayed with gold, and the bacterial morphology was observed using scanning electron microscope (SEM).

### Study on the regulation of macrophage phenotype

FCM was utilized to analyze the expression of the surface markers for M1 (CD86) and M2 (CD206) phenotypes. RAW 264.7 cells were seeded in 6-well plates at a density of 10^5^ cells per well and cultured overnight at 37 °C with 5% CO_2_. The cells were pre-treated with either HA@CUR NPs or CUR solution, both containing an equivalent amount of CUR (10 µg/mL). Cells were treated with 1 µg/mL LPS for 12 h. Subsequently, cells were washed thrice with PBS, centrifuged, and the supernatant discarded. Each tube received 1 mL of PBS containing 0.5% FBS (for FACS) for resuspension, followed by another centrifugation and supernatant removal. Cells were resuspended in 100 µL of FACS buffer and incubated at 4 °C for 5 minutes. Antibodies were added to the above solution, 5 µL CD86 and 5 µL F4/80 for M1 group and 5 µL F4/80 for M2 group, mixed well, protected from light, and incubated on ice for 20–30 min, during which it was blown 2–3 times. After centrifugation, the M1 group samples each received 2% paraformaldehyde (PFA) for fixation at room temperature for 20 min, followed by centrifugation and re-suspension in 300 µL FACS buffer. The M2 group underwent identical procedures as the M1 group, with an additional inclusion of 5 µL CD206 antibody, mixed thoroughly. Suspensions were filtered through a grid (40 μm pore size) and detected using a flow cytometer. The data obtained were used to complete the analysis of the results using CytExpert software (V 2.3).

### Effects on the expression of inflammatory factors in RAW264.7 macrophages

#### Study of RNA expression levels

RAW 264.7 cells were seeded at a density of 2 × 10^5^ cells per well in 6-well culture plates and incubated overnight at 37 °C with 5% CO_2_. After 12 h, the experimental groups received CUR solution or HA@CUR NPs solution. 5 hours later, LPS was added to the culture medium to a final concentration of 1 µg/mL. 4 h post-LPS treatment, the original culture medium was removed, and the cells were washed 3 times with PBS, followed by RNA extraction. The expression levels of TNF-α, IL-6, IL-1β, Mmp8, COX-2, Arg-1 and iNOS were detected by qPCR. The primer sequences are shown in Table S1.

#### Study of protein expression levels

Western Blot: RAW 264.7 cells were plated at a density of 2 × 10^5^ cells per well on 6-well culture plates and cultured overnight at 37 °C in a 5% CO_2_ incubator. After 12 h, the experimental groups were treated with either CUR or HA@CUR NPs solution. 5 hours later, LPS was added to achieve a final concentration of 1 µg/mL. After 24 h of LPS addition, the original culture medium was removed and the cells were lysed by adding 1X RIPA buffer and protein inhibitor mixture after 3 washes with PBS. Cellular proteins were quantified using a BCA protein assay kit. Cellular proteins were quantified using the BCA protein assay kit, then the supernatant containing 20 µg of protein was subjected to electrophoresis, and the proteins were transferred to a PVDF membrane, which was closed in using 5% skimmed milk powder or BSA and incubated with primary and secondary antibodies. The protein bands were visualized using an ECL chemiluminescence detection kit and ChemiDoc™ XRS + imaging system, with quantitative analysis of protein bands performed using Image J software (National Institutes of Health, Bethesda, MD, USA).

### Antioxidant assays

#### DPPH radical scavenging activity measurement

Different concentrations of CUR or HA@CUR NPs solutions were prepared first, and then DPPH radical reserve solution with a concentration of 1 mM was prepared with anhydrous ethanol and stored away from light. To 3.0 mL of ethanol solution of DPPH at a concentration of 0.1 mM was added 3.0 mL of different concentrations of CUR or HA@CUR NPS solution, shaken well and then immediately placed away from light for 1 h. Absorbance at 517 nm was measured by UV spectrophotometer. Each sample was repeated 3 times.

$$\eqalign{{\rm{DPPH}}\,{\rm{Radical}}\,{\rm{Scavenging}}\,{\rm{Activity}}\,\left( {\rm{\% }} \right)\,{\rm{ = }}\, & \cr & \left[ {\left( {{\rm{A0 - A1}}} \right){\rm{ / A0}}} \right]\,{\rm{ \times }}\,{\rm{100\% }} \cr}$$

Where: A0 is the optical density (OD) of the DPPH solution with anhydrous ethanol and A1 is the OD of the DPPH solution with the addition of the sample solution.

#### ABTS radical scavenging activity measurement

10 mL of 2 mM ABTS solution and 10 mL of 2.45 mM potassium persulfate was mixed homogeneously and kept away from light for 12–16 h as a stock solution. The ABTS radical stock solution was diluted with anhydrous ethanol before the reaction to give an absorbance of 0.700 ± 0.02. 1 mL of CUR or HA@CUR NPs solution at different concentrations was added to 4.5 mL of ABTS radical solution, shaken well, and then immediately placed away from light for 10 min. The absorbance at 734 nm was measured by UV spectrophotometer. Each sample was repeated 3 times.

$$\eqalign{{\rm{ABTS}}\,{\rm{Radical}}\,{\rm{Scavenging}}\,{\rm{Activity}}\,\left( {\rm{\% }} \right)\,{\rm{ = }} & \cr & \left[ {\left( {{\rm{A0 - A1}}} \right){\rm{ / A0}}} \right]\,{\rm{ \times }}\,{\rm{100\% }} \cr}$$

Where: A0 is the absorbance value of the ABTS solution with anhydrous ethanol and A1 is the absorbance value of the ABTS solution with the addition of the sample solution.

#### Ferric reducing antioxidant power (FRAP) Assay

The samples were diluted into different concentration gradients with distilled water. 1 mL of the sample was taken and added with 2.5 mL of PBS buffer solution (pH 6.6) as well as 2.5 mL of 1% potassium ferricyanide, and incubated at 50 ℃ for 30 min, and cooled in an ice bath immediately after the end of the incubation. Subsequently, 2.5 mL of 10% trichloroacetic acid was added, mixed well and centrifuged. Then 2.5 mL of supernatant was taken, and 2.5 mL of distilled water and 0.5 mL of 0.1% FeCl3 solution were added, and the reaction was carried out at 25 ℃ for 10 min, then the OD value at 700 nm was measured, and the higher the OD value was, the stronger the reducing ability and the better the antioxidant effect was.

### Antioxidant stress studies

RAW 264.7 cells were seeded in a 96-well plate at a density of 2000 cells/well, with 100 µL of DMEM complete culture medium added to each well, and incubated overnight at 37 °C with 5% CO_2_. The original medium was aspirated, and medium containing various concentrations of H_2_O_2_ was added to the wells with six replicate wells per group, followed by a further incubation for 24 h. After 24 h, the original culture medium was aspirated, washed 3 times with PBS, 100 µL of serum-free medium and 10 µL of CCK8 solution were added, and the cells were incubated at 37 ℃ in a 5% CO2 incubator for 3 h. Finally, the absorbance at 450 nm was measured by a multifunctional enzyme marker, and the cell survival rate was calculated.

RAW 264.7 cells were plated at a density of 2000 cells/well in a 96-well plate and incubated overnight at 37 °C with 5% CO_2_. After 12 h, the original medium was discarded, and the experimental groups were treated with a culture medium containing 10 µg/mL of either CUR or HA@CUR NPs for an additional 5 h. Both control and experimental groups were then challenged with H_2_O_2_ (final concentration: 1600 µM) for 24 h under the same incubation conditions. The original culture medium was removed, cells were washed 3 times with PBS, and each well received 100 µL of serum-free culture medium plus 10 µL of CCK8 solution. After incubating for another 3 h at 37 °C, absorbance at 450 nm was measured using a multifunctional enzyme marker to calculate the survival rate of each group of cells.

### Effects on antioxidant gene expression of HO-1, CAT, and SOD

RAW 264.7 cells were seeded at a density of 2 × 10^5^ cells/well in a 6-well plate and incubated overnight at 37 °C with 5% CO_2_. After 12 h, the experimental groups were treated with solutions of CUR or HA@CUR NPs, followed by the addition of H_2_O_2_ to a final concentration of 800 µM in the culture medium after 5 hours. 4 h post-H_2_O_2_ treatment, the original medium was removed, the cells were washed 3 times with PBS solution, and then RNA was extracted. The expression levels of the HO-1, SOD, and CAT genes were detected by quantitative PCR (qPCR). Primer sequences are listed in Table S1.

### Intracellular ROS scavenging activity

RAW 264.7 cells were plated at a density of 2 × 10^4^ cells/well into laser confocal dishes and cultured overnight at 37 °C with 5% CO_2_. The original medium was removed, and experimental groups were treated with media containing either CUR or HA@CUR NPs, while the blank and control groups received media with PBS, continuing incubation for 12 h. The control and experimental groups were then challenged with an equal volume of H_2_O_2_ to induce cellular stress, achieving a final H_2_O_2_ concentration of 800 µM, and incubated for 2 h. Following this, the original medium was replaced with serum-free medium containing DCFH-DA and incubated in the dark at 37 °C for 0.5 h. After washing 3 times with PBS, cells were observed and photographed under a laser-scanning confocal microscope.

To further investigate the intracellular ROS scavenging activity, FCM was employed. RAW 264.7 cells were seeded at a density of 2 × 10^4^ cells/well in a 6-well plate and treated using the same protocol as mentioned above. Cells were then collected, washed and centrifuged, and finally resuspended in PBS liquid containing 2% FBS. The suspension was filtered through a grid (40 μm) and DCFH-DA fluorescence was detected by FCM.

### Establishment of a chronic periodontitis model in vivo

All animal procedures were conducted in accordance with the guidelines set by the Animal Care and Use Committee of Chongqing Medical University. To establish a chronic periodontitis model, 6-week-old male Sprague-Dawley rats weighing 200–250 g were fasted for 12 h and then anesthetized with enflurane gas. Chronic periodontitis was induced by ligating the maxillary first molar with a 0.2 mm diameter stainless steel wire, which was inserted into the gingival sulcus. After 4 weeks, the ligature was removed, and the rats were treated with CUR or HA@CUR NPs for an additional 4 weeks. Drug administration was carried out every other day by subgingival injection at six sites around each tooth, with 10 µL injected per siteRats without any treatment were blank group and ligation treated rats were control group. Both blank and control groups were injected with equal amount of PBS solution. rats were executed after 4 weeks and tissues were collected for analysis.

### Animal imaging analysis

After 4 weeks of treatment, ROS levels were detected by local in situ injection of the DCFH-DA probe. Fluorescence imaging was performed on a small animal imaging system (*n* = 3) to measure and quantify fluorescence signal intensity. In addition, local in situ injections of HA-Cy5.5 NPs and free Cy5.5 were performed using fluorescence imaging on an animal imaging system (*n* = 3), and fluorescence signal intensities were measured and quantified to detect the retention of nanoparticles over time in animals.

### Micro-CT analysis

All harvested maxillary bone samples were fixed overnight in 4% paraformaldehyde (PFA) and subsequently scanned using a Scanco µCT50 scanner (Scanco Medical AG, Brüttisellen, Switzerland) at a resolution of 18 μm, with a scanning voltage of 70 kV and a scanning current of 200 µA. Three-dimensional reconstructions and two-dimensional X-ray images were generated using SCANCO Visualizer software. Alveolar bone loss (ABL) was determined by measuring the distance from the cementoenamel junction (CEJ) to the alveolar bone crest (ABC). All measurements were carried out using ImageJ software. Moreover, the region of interest (ROI) for the analysis of bone-related parameters was selected between the mesial side of the first molar and the distal side of the second molar at the medial area. We constructed the ROI starting from the slice where the CEJ first appeared, extending downwards to the level where the distal root apex of the first maxillary molar first disappeared. Each sample consisted of 30 layers of ROI. The ROI percentage of bone volume (BV/TV), trabecular separation (Tb.sp), trabecular number (Tb.N), and trabecular thickness (Tb.Th) within the affected bone tissue were recorded using SCANCO Evaluation software. Furthermore, major organs including the heart, liver, spleen, lung, and kidney were stained with hematoxylin and eosin (H&E) for toxicity analysis. The sections were scanned and photographed using a microscope.

### RT-PCR of rat periodontal tissues

The rat periodontal tissue was homogenized and centrifuged using Trizol reagent. The supernatant was removed, chloroform and trizol was added, incubated for 5 min at room temperature and centrifuged at 12,000 rpm. The topmost layer was taken and mixed with isopropanol trizol and 75% ethanol. The mixture was vortexed, centrifuged, supernatant removed and air dried to obtain an RNA pellet. RNA was eluted with 20 µL RNase-free water. Finally, the concentration of RNA was determined by Nano drops Thermo scientific 2000 spectrometer. The RNA was then converted to cDNA by reverse transcription, and the expression of rat genes was detected by real-time fluorescence quantitative polymerase chain reaction (qRT-PCR).

Isolation of bacterial RNA by differential lysis:

(i) Sample preparation: Periodontal tissues from each group of rats were excised and immediately placed in Petri dishes containing 2 mL Tris EDTA (TE) buffer (10 mM Tris-HCl, 1 mM EDTA). Periodontal tissue was cut into smaller pieces, transferred to EP tubes, and broken up with a tissue eruptor (Qiagen). 4 mL of TE buffer was added and the tissue homogenates were digested with 1 ml of 20 mg/mL proteinase K solution (Qiagen) at 55 ℃ for 10 min (without shaking). Another 6 mL of TE buffer was added and the samples were centrifuged at 3200 g for 15 min at 4 ℃. Microspheres containing bacterial cells were resuspended with 300 µL of Tri-reagent reagent (Zymo Research) containing 1% β-mercaptoethanol and 300 µL of RLT buffer (Qiagen).
(ii) RNA extraction and purification: samples were transferred into bead tubes (0.1 mm glass beads, MoBio) and shaken using a TissueLyser (Qiagen) at 30 Hz for 5 min at 4 ℃. Samples were immediately placed on ice and centrifuged at 10,000 g for 5 min at 4 ℃ to remove cellular debris. The supernatant was transferred to a clean tube containing 1 volume of 100% ethanol and mixed by repeated gentle inversion.

The primer sequences are shown in Table S2 and Table S3.

### Histological and Immunohistochemical Analysis

Maxillary bone tissue sections were decalcified in a 10% solution of ethylenediaminetetraacetic acid (EDTA) for two months, with a change of decalcification fluid every two days. Subsequently, samples were dehydrated and embedded in paraffin. Sections were cut to a thickness of 5 μm in a mesiodistal orientation using a microtome. Sections were stained with H&E, tartrate-resistant acid phosphatase (TRAP), and immunohistochemically for inflammatory cytokines (IL-6, IL-1β, and TNF-α), as well as antioxidants (HO-1, SOD, and CAT) to observe anti-inflammatory, antioxidant, and osteoclastogenesis inhibitory effects among the different treatment groups.

### Statistical analysis

Statistical differences between groups were determined using independent two-sample t-tests or one-way analysis of variance (ANOVA). Data are presented as mean ± SD. Statistical significance was defined as p* < 0.05, p** < 0.01, p*** < 0.001.

## Results and discussion

**Fig. 1:**
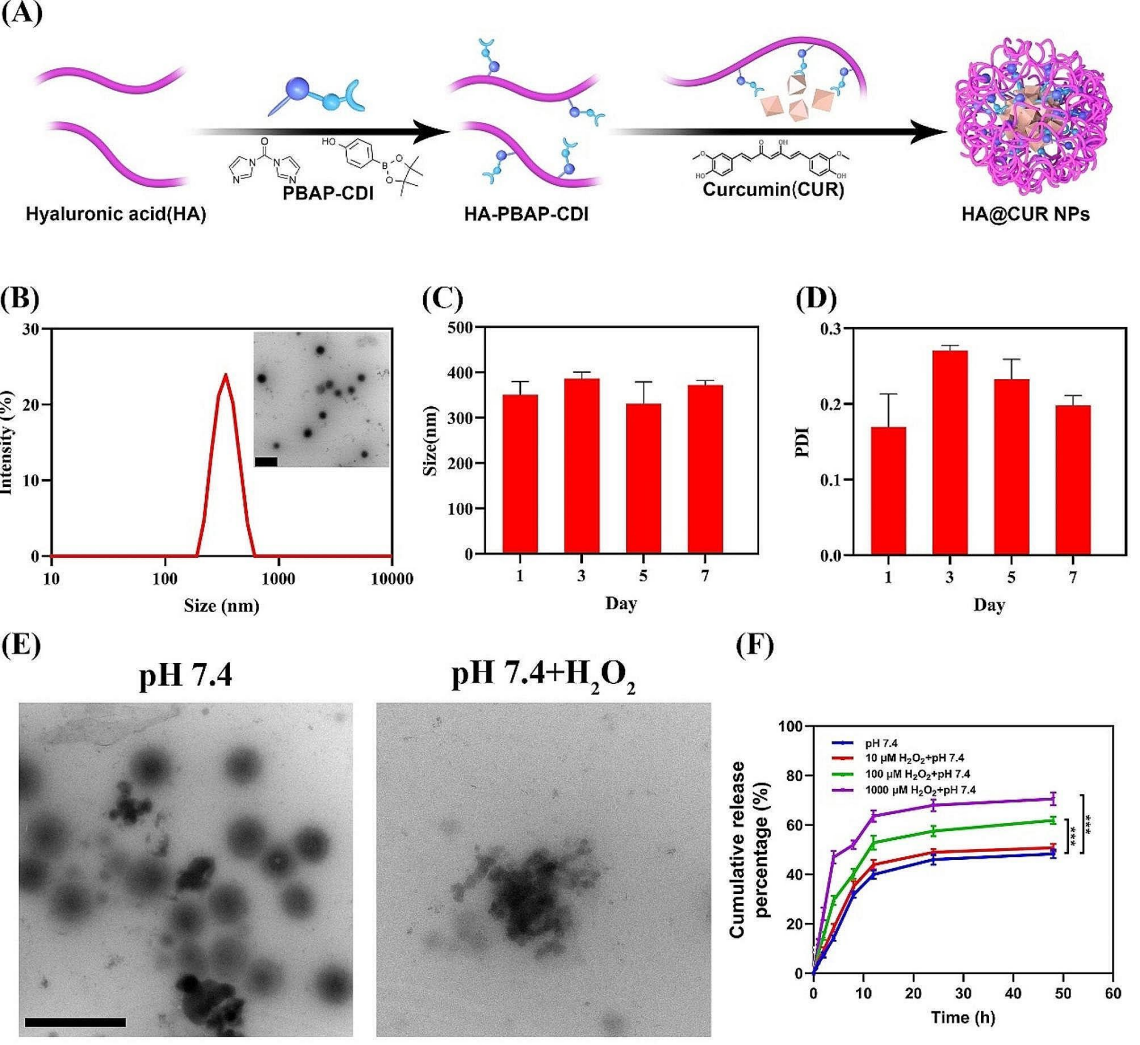
Preparation and Characterization of HA@CUR NPs. (**A**) Illustrative schematic of the preparation process for HA@CUR NPs; (**B**) TEM images depicting the morphology and aqueous particle size distribution of HA@CUR NPs (Bar = 1 μm); (**C**) Variation in particle size of HA@CUR NPs over the course of 1, 3, 5, and 7 days of storage at room temperature, and (**D**) changes in the dispersity coefficient; (**E**) TEM images of HA@CUR NPs before and after the addition of H_2_O_2_. (Bar = 1 μm); (**F**) Release rate profiles of HA@CUR NPs in the different concentrations of H_2_O_2_. (*n.s.*: not significant; *p < 0.05; **p < 0.01; ***p < 0.001)

The macromolecular structure of HA contains many modifiable reactive groups, such as carboxyl and hydroxyl groups. These groups can be used to prepare HA derivatives with different functions *via* esterification, amidation, grafting and other methods [22]. Here, using CDI as an intermediary, PBAP was grafted onto the main HA backbone to synthesize a ROS-responsive material. The structural characterization of the material can be observed *via* FT-IR and ^1^H-NMR (Figure S1, S2). Subsequently, HA@CUR NPs were formulated through self-assembly with CUR in water (Fig. 1A). The particle size of HA@CUR NPs in water was 417.16 ± 48.67 nm (Fig. 1B), with a zeta potential of -57.47 ± 0.18 mV (Figure S3). In a normal physiological environment, HA@CUR NPs predominantly displayed a spherical morphology (Fig. 1B) and exhibited good aqueous stability. As indicated in (Fig. 1C and D), HA@CUR NPs maintained their initial particle size throughout the experimental process, and the PDI remained below 0.3 in the aqueous medium. In order to clarify the interaction force between CUR and materials and the existence form of CUR in NPs in HA@CUR NPs, FTIR spectroscopy was used to demonstrate the formation of HA@CUR NPs. In addition, molecular interactions between free CUR and the NPs were characterized to verify successful encapsulation of CUR by the drug carrier. FTIR for free CUR, blank NPs, and CUR-loaded NPs are shown in (Figure S4). In the spectrum of CUR, characteristic absorption peaks for -OH, C = O, and C = C stretching vibrations appeared at 3422 and 1723 cm^− 1^, respectively, while phenyl skeletal vibrations of the CUR phenyl ring were at 1508 and 1497 cm^− 1^ [23, 24]. When CUR was encapsulated within the NPs, characteristic peaks of CUR at 1723, 1508, and 1497 cm^− 1^ shifted to 1728, 1521, and 1501 cm^− 1^ respectively. It indicated that there was an interaction between the CUR and the material. On the other hand, XRD was employed to characterize the physical state of CUR within the NPs. From the results (Figure S5), free CUR exhibited sharp and distinctive crystalline diffraction peaks, implying a highly crystalline state for free CUR. In contrast, when CUR was encapsulated in NPs, the crystalline diffraction peaks of free CUR disappeared and only characteristic peaks similar to those of the material were present. This shows that CUR exists mainly in the NPs in an amorphous or free state [25, 26], which helps the drug to leach out of the carrier during release.

The ROS-responsiveness of PBAP, the spherical morphology of HA@CUR NPs exhibited changes upon the addition of H_2_O_2_. During this process, the NPs progressively fragmented until they were completely ruptured (Fig. 1E). Furthermore, the particle size distribution of HA@CUR NPs displayed two distinct ranges in the presence and absence of H_2_O_2_. Monitoring the PDI on day 1 and day 3 revealed that the PDI of HA@CUR NPs increased over time in the H_2_O_2_ environment (Figure S6). To further investigate the ROS-responsive drug release characteristics of HA@CUR NPs, we studied the in vitro release rate of CUR in PBS with different concentration of H_2_O_2_. As shown in Fig. 1F, there was no significant difference in drug release from HA@CUR NPs within the first 0.5 h (approximately 5%) in the all group. After 4 h, a significant difference emerged in the cumulative drug release from HA@CUR NPs in different media (14.34 ± 1.11, 18.38 ± 1.63, 29.55 ± 1.93, and 46.99 ± 2.51% in the 0, 10, 100, and 1000 µM of H_2_O_2_, respectively) indicating that the drug release from HA@CUR NPs is ROS-responsive. After 48 h, the cumulative drug release rate of HA@CUR NPs in the 100 and 1000 µM of H_2_O_2_ reached 61.85 ± 1.42 and 70.53 ± 2.54%, which was significantly higher than the 48.29 ± 1.76% observed without H_2_O_2_. These results confirm that HA@CUR NPs possess favorable ROS-responsive release characteristics. For drug carriers, a lower drug release rate in a normal physiological environment is beneficial for reducing adverse side effects. At the same time, ROS-triggered drug release ensures effective concentrations in the therapeutic window during lesion treatment. Summary, CUR NPs demonstrated ROS-responsive release efficiency, potentially enhancing the treatment efficacy for periodontitis.

On the other hand, biosafety is an important consideration for the further applications of drug delivery systems. In this context, RAW264.7 cells and HGF cells were selected as model cell lines for the study. As indicated by the results (Figure S7), both free CUR and HA@CUR NPs showed no impact on cell viability at all tested concentrations, demonstrating good cytocompatibility for both. In addition, the hemolytic toxicity of HA@CUR NPs was also assessed, and results showed that at all tested concentrations. HA@CUR NPs did not exhibit any significant hemolytic toxicity (Figure S8). These findings confirm that HA@CUR NPs possess not only good cytocompatibility but also favorable hemocompatibility.

### Cell uptake study

**Fig. 2:**
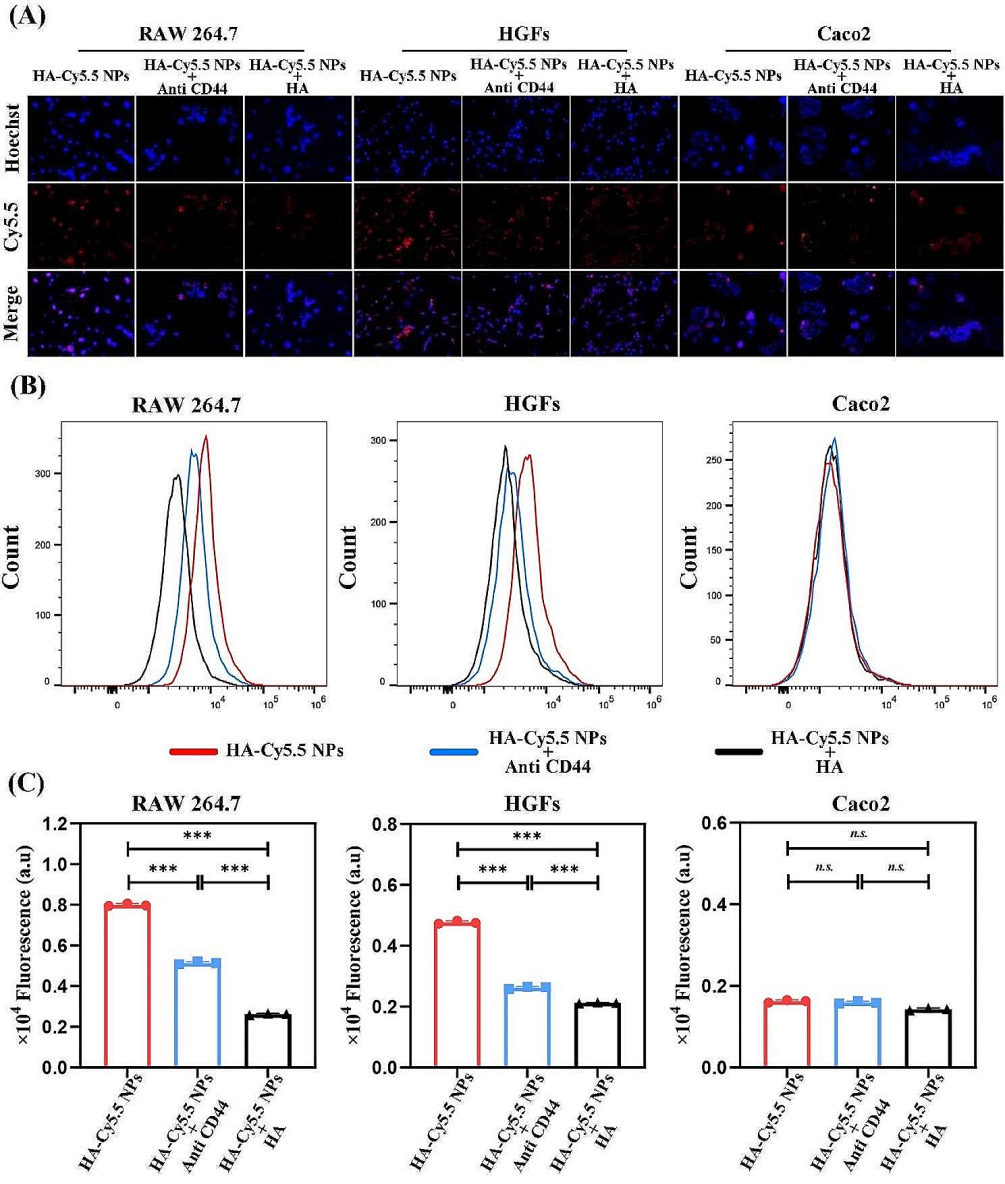
(**A**) Fluorescent microscopic observation (blue: Hoechst, red: Cy5.5) of RAW 264.7, HGFs and Caco2 uptake of HA-Cy5.5 NPs before and after treatment with anti CD44 and HA; (**B**) FCM and (**C**) quantitative analysis of RAW264.7 cell uptake of RAW 264.7, HGFs and Caco2 uptake of HA-Cy5.5 NPs before and after treatment with anti CD44 and HA. (*n.s.*: not significant; *p < 0.05; **p < 0.01; ***p < 0.001)

CD44 is a type I transmembrane protein that contains three structural domains, including an extracellular domain, a transmembrane domain and an intracellular domain, and is normally found in normal cells [27]. HA, as a naturally-occurring mucopolysaccharide, has a primary receptor for CD44, and is therefore widely used as a therapeutic targeting vehicle. To investigate the effect of CD44 on HA-based carrier uptake, cells with high expression of CD44 (e.g. RAW 264.7 and HGFs) and low expression of CD44 (e.g. Caco2) were selected for the study. To demonstrate the targeting of HA-based nanocarriers in relation to CD44 abundance, we assessed the expression levels of CD44 in RAW 264.7, HGFs and Caco2 cells by FCM. The results showed that both RAW 264.7 and HGFs expressed high levels of CD44, whereas Caco2 had little CD44 expression (Figure S9). To observe cellular uptake, fluorescent Cyanine 5.5 (Cy5.5) labeling was introduced on HA-based nanocarriers. A significant increase in red signal was observed in CD44 highly expressing cells RAW264.7 and HGFs (Fig. 2A). This contrasted with the use of pretreatment groups by CD44 antibody or HA, indicating disrupted fluorescence uptake. The data suggest that CD44 plays a key role in HA-based nanocarrier uptake. Experiments were performed with the addition of CD44 low expressing Caco2 cells, in which there was no significant cellular uptake. Upon addition of anti CD44 or HA, no significant change in the uptake of nanocarriers by this cell was seen (Fig. 2A). To further confirm the enhanced uptake of HA-based nanocarriers in relation to CD44 abundance, FCM was also utilized in the study. FCM results showed (Fig. 2B and C) that cellular more uptake by high-expressing CD44 cells (RAW264.7 and HGFs) than low expressing cells (Caco2).

**Fig. 3:**
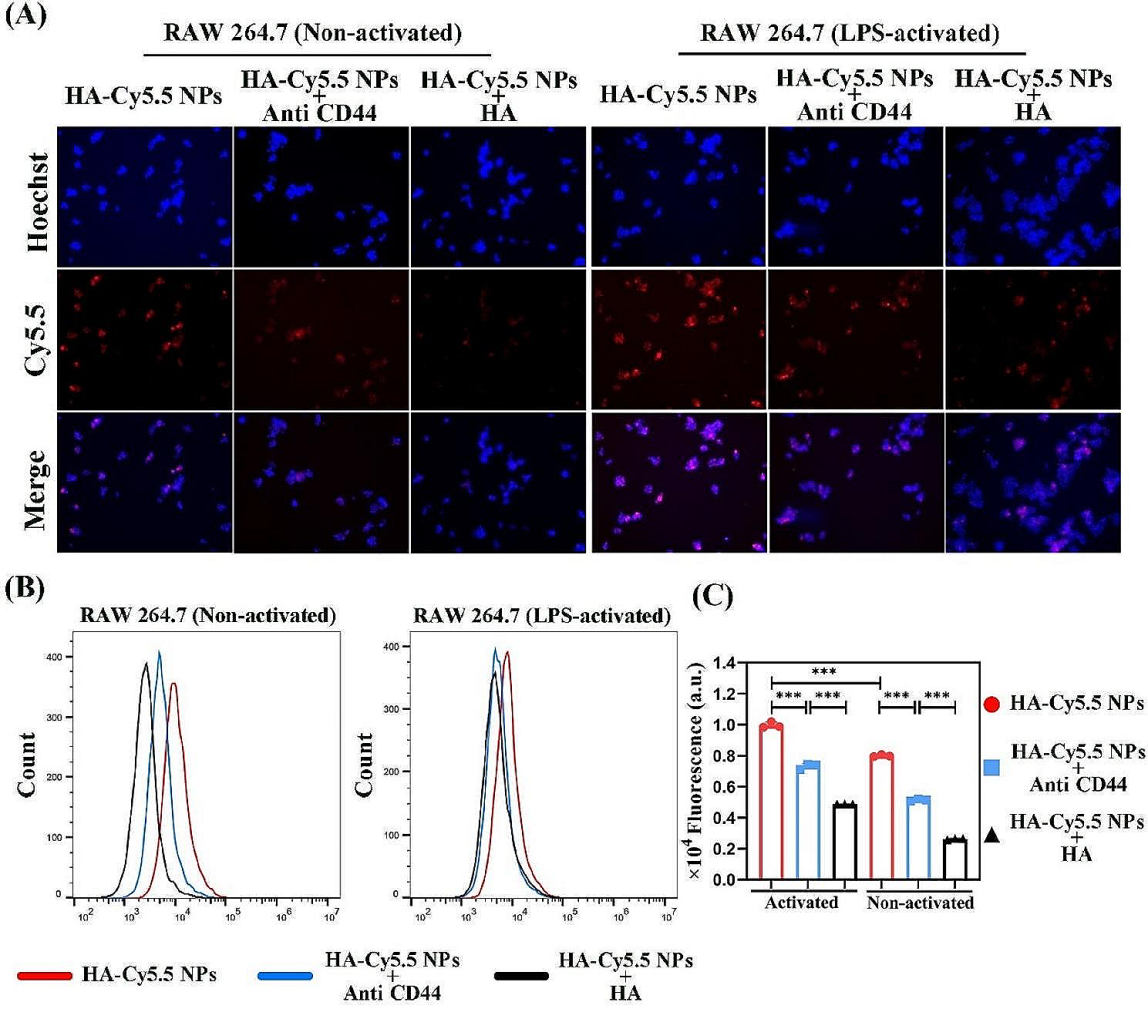
(**A**) Fluorescent microscopic observation (blue: Hoechst, red: Cy5.5) for non-activated RAW264.7 and activated RAW 264.7 uptake of HA-Cy5.5 NPs before and after treatment with anti CD44 and HA. (**B**) FCM and (**C**) quantitative analysis for non-activated RAW 264.7 and activated RAW 264.7 uptake of HA-Cy5.5 NPs before and after treatment with anti CD44 and HA. (*n.s.*: not significant; *p < 0.05; **p < 0.01; ***p < 0.001)

Macrophages represent one of the most prevalent cellular components within the inflammatory milieu, perpetuating the production of inflammatory mediators during host response dysregulation, which results in severe tissue damage and contributes to the onset and progression of various diseases. Significantly, macrophages are innate immune cells located on the epithelial surfaces of periodontal tissues, exhibiting differential responses to various stimuli from pathogens and commensal bacteria and are key cells in the pathophysiology of chronic inflammation [14]. Hence, in this study, RAW 264.7 cells were activated by LPS or not as model cells for investigating cellular uptake. From the results (Fig. 3A), the activated-RAW 264.7 was able to uptake more HA-Cy5.5 NPs and had a stronger fluorescent signal than the non-activated RAW 264.7. Subsequently, further detection was carried out by FCM. The FCM results showed (Fig. 3B, 3 C) that stronger fluorescence signals were visible in the activated cells compared to the non-activated RAW 264.7. This result may be related to elevated levels of CD44 before and after macrophage activation [20].The aforementioned results indicate HA does have the potential as a nanoparticle encapsulating material to enhance uptake by cells of CD44 high expression. HA-based nanocarrier achieve specific targeting abilities based on the interaction between HA and CD44, ensuring effective retention at the cellular level. This provides a certain level of assurance for improving therapeutic efficacy in the treatment of periodontitis.

### In vitro antimicrobial experiment

**Fig. 4:**
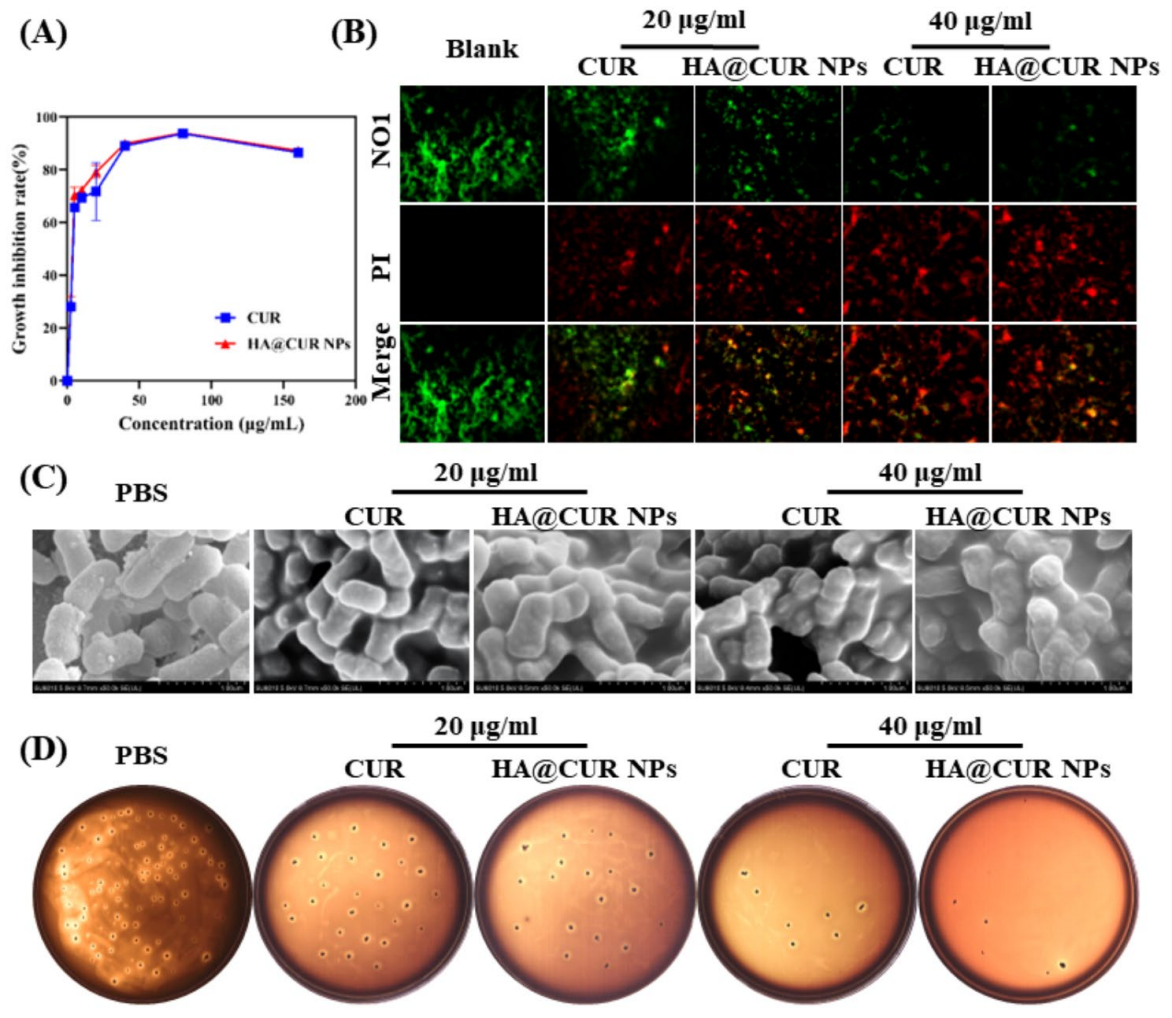
Inhibitory and bactericidal effects of HA@CUR NPs on *P.g.*; (**A**) Inhibition rate of *P.g.* by different concentrations of CUR solution and HA@CUR NPs; (**B**) Live/Dead staining of *P.g*. biofilms following the addition of HA@CUR NPs and CUR at equivalent drug concentrations; (**C**) Representative scanning electron microscopy images of *P.g.* following treatment with PBS, HA@CUR NPs, and CUR at the same drug concentrations; (**D**) Colony formation experiments of *P.g.* after treatment with PBS, HA@CUR NPs, and CUR at equivalent drug concentrations

Periodontitis is caused by the interaction of pathogenic microorganisms with various immune cells in the tissues [14]. CUR, as a natural drug with antimicrobial properties, also plays a role in the elimination of pathogenic microorganisms in the treatment of periodontitis [28]. *P.g.* was selected as the model bacterial strain for the study of the antimicrobial activity of HA@CUR NPs. The results (Fig. 4A) indicate that HA@CUR NPs, like free CUR, exhibit noticeable bactericidal effects. Statistical analysis shows that the minimum inhibitory concentration (MIC) values for both groups are consistent, at 40 µg/mL. Notably, at 20 µg/mL, HA@CUR NPs exhibit slightly superior inhibition of *P.g.* compared to the free CUR treatment group. Overall, HA@CUR NPs inherit CUR’s antibacterial capability.

On the other hand, bacteria can form an organized cellular colony known as a biofilm. Biofilms provide a stable environment for microbes, complicating drug treatment and exacerbating the condition [29]. This study investigated the inhibitory effect of HA@CUR NPs on biofilms using a live/dead staining method. The results (Fig. 4B) demonstrate that HA@CUR NPs and free CUR exhibit significant concentration-dependent inhibition of biofilms. With increasing drug concentration, there was a noticeable decrease in green fluorescent signals and a marked increase in red fluorescence signals. Compared to the CUR treatment group, HA@CUR NPs exhibited stronger biofilm inhibition at both high and low concentrations. These benefit from HA itself has some antimicrobial activity, and the antimicrobial capacity of the nanoparticles is enhanced after CUR is encapsulated by HA to form nanoparticles.

To visually demonstrate the antimicrobial efficacy of HA@CUR NPs against *P.g.*, SEM was utilized to observe the morphologies of *P.g.* in various treatment groups (Fig. 4C). The results show that compared to the PBS-treated group, the membrane surfaces of *P.g.* in the HA@CUR NPs treatment group displayed signs of collapse and shrinkage at both low and high concentrations. In contrast, free CUR treatment only caused significant killing at high concentrations. To further demonstrate the antibacterial effects of each group, blood agar plating cultures for *P.g.* were performed. Figure 4D reveals that both HA@CUR NPs and free CUR exhibited a concentration-dependent colony suppression of *P.g.*. Notably, the bacterial count for the HA@CUR NPs treatment group was lower than that of the free CUR treatment group at both high and low concentrations.

These results confirm that HA@CUR NPs possess good antimicrobial activity and exhibit a significant inhibitory effect on biofilms formed by *P.g.*, showing potential as an antimicrobial agent in the treatment of periodontitis.

### In vitro antioxidant experiments

**Fig. 5:**
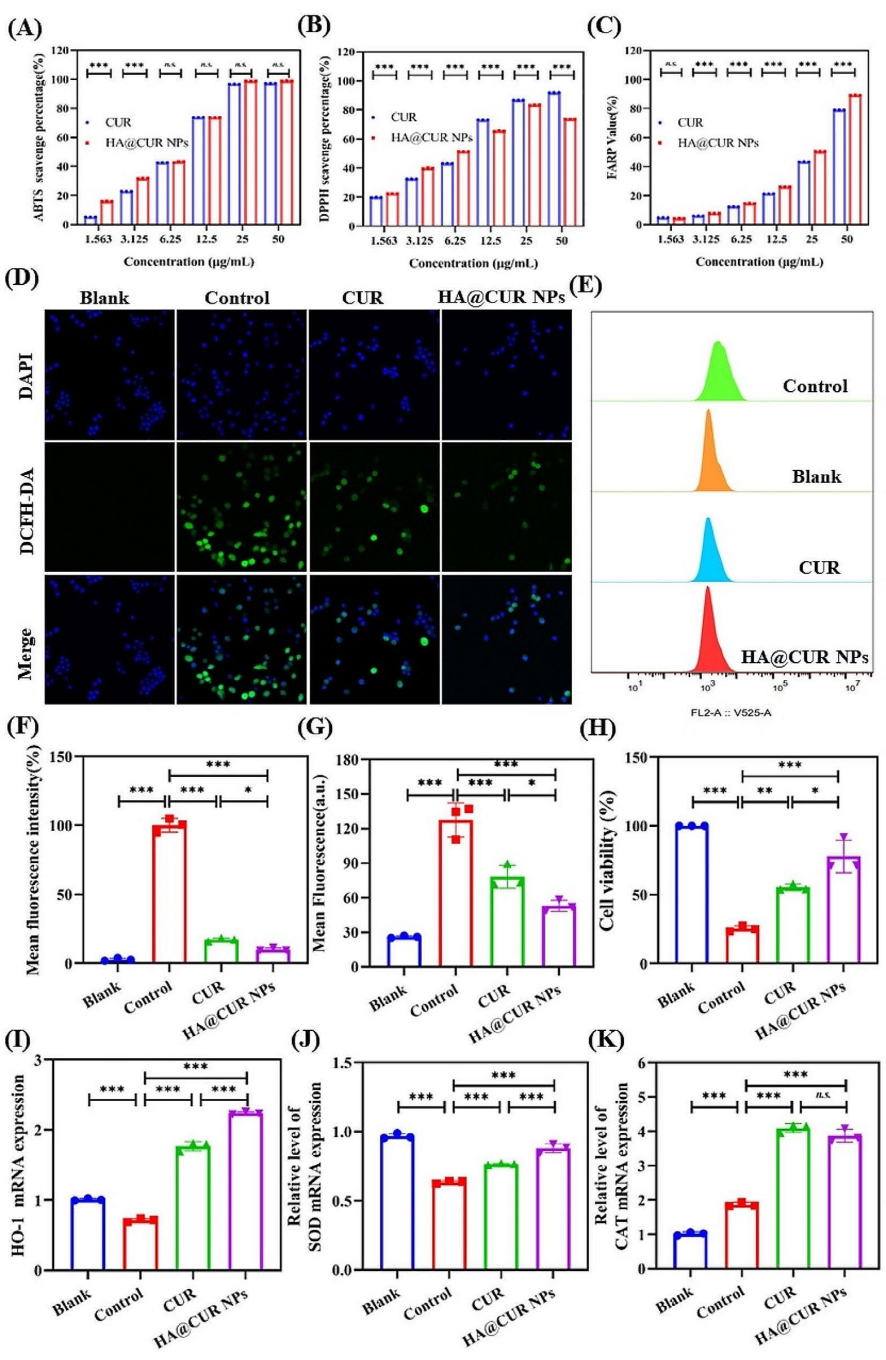
In vitro antioxidant capacity of HA@CUR NPs. (**A**) The free radical scavenging activity of HA@CUR NPs and CUR against DPPH and (**B**) ABTS radicals, and (**C**) the iron reduction ability assessment. (**D**, **E**) The capacity of equivalent concentrations of HA@CUR NPs and CUR to clear intracellular ROS as observed under laser confocal microscopy and their quantitative analysis. (**F**) FCM detection of ROS clearance in RAW 264.7 cells treated with H_2_O_2_ by HA@CUR NPs and CUR, and (**G**) the quantification of fluorescence intensity. (**H**) Protective effects of HA@CUR NPs and CUR on RAW264.7 cells stimulated with a solution of 1600 µM concentration of H_2_O_2_, where both increased cell viability. (**I-K**) HA@CUR NPs and CUR enhance the gene expression of antioxidative factors HO-1, SOD, and CAT. (n = 3) (*n.s*.: not significant; *p < 0.05; **p < 0.01, and ***p < 0.001)

Periodontitis is defined as a chronic inflammatory disease wherein the persistent activation of oxidative stress exceeds the scavenging ability of the endogenous antioxidant defense system to clear ROS [30, 31]. Therefore, the elimination of excess ROS in periodontal tissues plays a crucial role in the treatment of periodontitis. Initially, we investigated the scavenging activity of HA@CUR NPs towards different free radicals, specifically DPPH and ABTS, in vitro. We also examined the antioxidant activity of HA@CUR NPs by assessing their iron-reducing capability. The results indicate (Fig. 5A, B and C) a concentration-dependent radical scavenging ability for both HA@CUR NPs and free CUR. The free radical scavenging capacities of the various formulations increased with the concentration. Overall, HA@CUR NPs exhibited relatively higher capabilities in scavenging free radicals compared to free CUR. On the other hand, although both formulations displayed a concentration-dependent trend in iron reduction, the iron-reducing capabilities of HA@CUR NPs were slightly superior to the free CUR group. A comprehensive evaluation of the data confirms that iron-reducing ability has been effectively preserved. These findings suggest that both HA@CUR NPs and free CUR possess antioxidative properties.

To investigate the capability of HA@CUR NPs and free CUR to clear cellular ROS, RAW 264.7 cells treated with H_2_O_2_ were chosen for study. Imaging results showed (Fig. 5D) that the fluorescence signal was significantly reduced in the HA@CUR NPs group and the free CUR group, compared to the control group. It indicated that the two formulations were both effective in scavenging intracellular ROS. Notably, the green signal in the HA@CUR NPs group was lower than that in the free CUR-treated group. The quantitative analysis of fluorescence from the images further confirmed this observation (Fig. 5E). To corroborate the ability of HA@CUR NPs and free CUR to eliminate ROS, FCM was used. The results revealed that HA@CUR NPs and free CUR could indeed reduce excessive cellular ROS (Fig. 5G). HA@CUR NPs showing greater ROS clearance abilities than the free CUR. Hence, it is apparent that HA@CUR NPs hold significant potential for enhancing macrophage uptake and thereby amplifying cellular ROS clearance capabilities.

Conversely, excessive ROS can cause damage to periodontal tissue cells, which may be a pivotal issue in treating periodontitis. Based on this, we verified the ability of two CUR formulations to resist oxidative damage in RAW 264.7 cells. As shown in Fig. 5H, free CUR exhibited significant antioxidative damage activity. However, HA@CUR NPs treatment showed stronger antioxidative damage activity, compared to free CUR. This is linked to some extent to the increase in CUR solubility by HA as well as increased cellular uptake.

Finally, we incorporated the detection of gene expression levels for heme oxygenase-1 (HO-1), superoxide dismutase (SOD), and catalase (CAT). The outcomes (Fig. 5I, J and K) demonstrated that compared with the control group, HA@CUR NPs and CUR could upregulate gene expression of antioxidative factors HO-1, SOD, and CAT. Furthermore, HA@CUR NPs had a more pronounced effect on enhancing the expression of HO-1 and SOD than free CUR. In summary, HA@CUR NPs are not only effective at inhibiting the production of free radicals but also markedly reduce intracellular ROS levels and protect against oxidative damage. Hence, nano-systems possessing ROS scavenging functions hold promise for ameliorating oxidative stress conditions in diseased tissues and more effectively treating periodontitis.

### Anti-inflammatory study

**Fig. 6:**
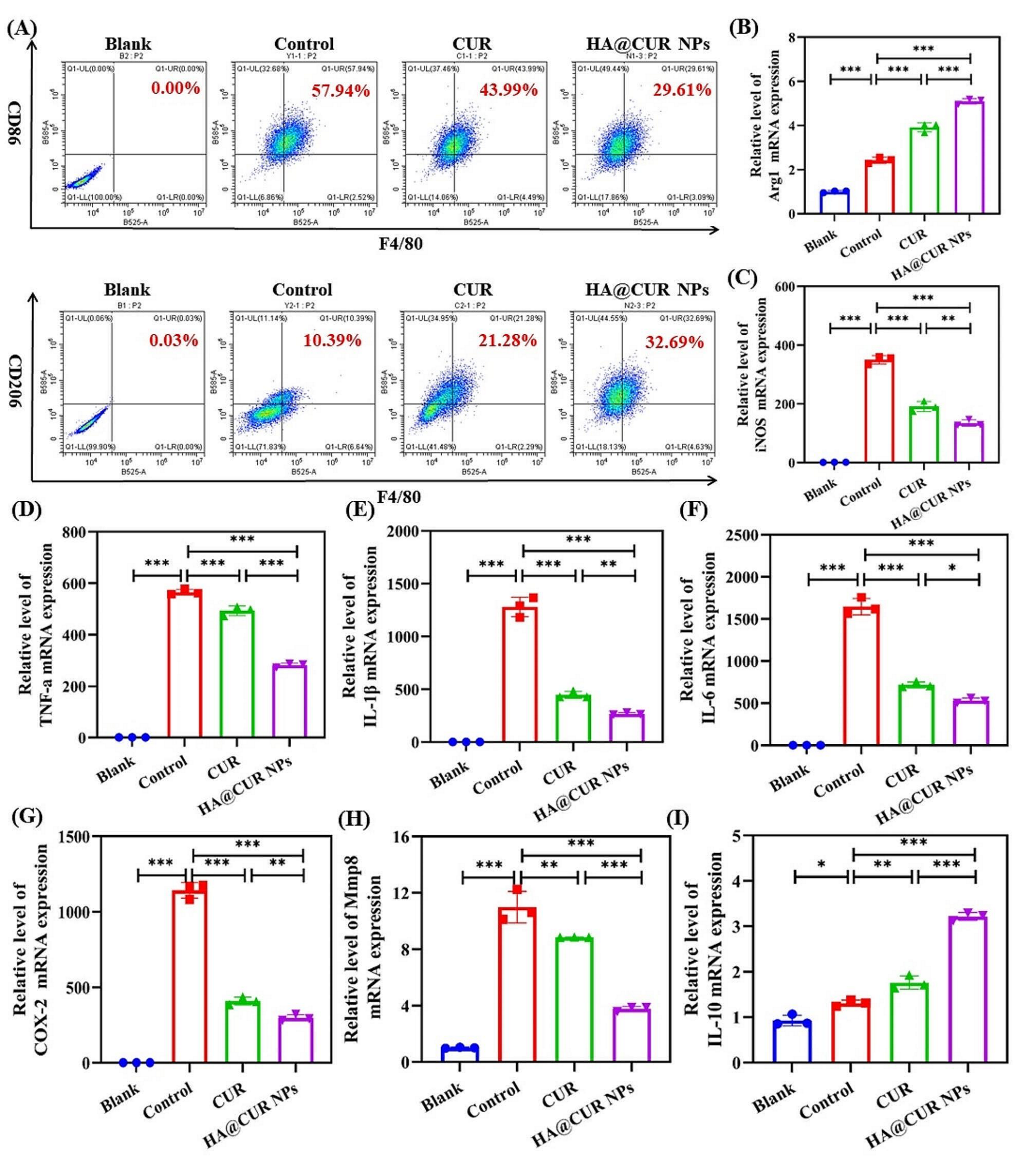
In vitro study of the effects of HA@CUR NPs and CUR on RAW 264.7 cell phenotypic transformation and gene expression of inflammatory factors under LPS stimulation. (**A**) FCM was used to assess the influence of HA@CUR NPs and CUR on the ratio of M1 to M2 macrophage phenotypes after LPS stimulation. CD86 (red, in the PE channel) and CD206 (red, in the PE channel) were used to specifically mark M1 and M2 type macrophages, respectively (antibody F4/80 labeled all macrophages, green, in the FITC channel). qPCR results of M1 macrophage marker gene (**B**) Arg1 and M2 macrophage marker gene (**C**) iNOS expression after RAW 264.7 cells were pre-treated with HA@CUR NPs and free CUR. (**D**) The gene expression levels of inflammatory factors (TNF-α, IL-1β, IL-6, Mmp8, COX-2). (*n* = 3) (*n.s.*: not significant; *p < 0.05; **p < 0.01; ***p < 0.001)

Periodontitis is an inflammatory disease, where the polarization of macrophages is in a dynamic equilibrium during inflammation development. M1 and M2 represent the two extreme phenotypes of the pro-inflammatory and anti-inflammatory functions in the macrophage polarization spectrum [14]. Therefore, the regulation of the M1/M2 ratio plays an important role in the health of periodontal tissue. Based on this, the study investigated the alleviation of inflammation by quantifying the ratio of M1 to M2 macrophages after treatment with different CUR formulations. Results indicated that free CUR and HA@CUR NPs groups were able to effectively increase the proportion of M2-type macrophages (Fig. 6A). The M1/M2 ratio was lower in the HA@CUR NPs group compared to free CUR. This suggests that HA@CUR NPs can effectively elevate the number of M2-type macrophages and reduce the number of M1-type macrophages. To further confirm this phenomenon, the study analyzed the gene expression levels of the M1 marker Arg1 and the M2 marker iNOS in Raw 264.7 cells pre-treated with HA@CUR NPs and free CUR (Fig. 6B and C). The results showed that the expression level of iNOS gene in the HA@CUR NPs group was higher than that in the control and free CUR groups. Conversely, Arg1 gene expression was significantly lower than that in the control and free CUR groups. These results suggest that HA@CUR NPs can regulate macrophage polarization to a certain extent. It is essential for modulating the M1/M2 ratio at periodontal inflammatory sites to alleviate the progression of inflammation in periodontal tissues.

On another note, CUR possesses anti-inflammatory properties [32, 33]. Here, by quantifying mRNA expression levels of TNF-α, IL-1β, IL-6, Mmp8, COX-2, and IL-10 in RAW 264.7 cells after treatment with different CUR formulations, the study observed the mitigation of inflammation. Results shown (Fig. 6D) that free CUR and HA@CUR NPs could effectively suppressed mRNA expression levels of TNF-α, IL-1β, IL-6, Mmp8, and COX-2. Simultaneously, the two formulations were able to increase mRNA expression levels of the anti-inflammatory cytokine IL-10. Compared with free CUR, the HA@CUR NPs group showed a greater reduction in the mRNA expression levels of inflammatory factors, e.g. TNF-α, IL-1β, IL-6, Mmp8 and COX-2. This may be attributable to enhanced uptake of HA@CUR NPs by macrophages and increased solubility of CUR.

### Treatment of ligature-induced rat periodontitis model

**Fig. 7:**
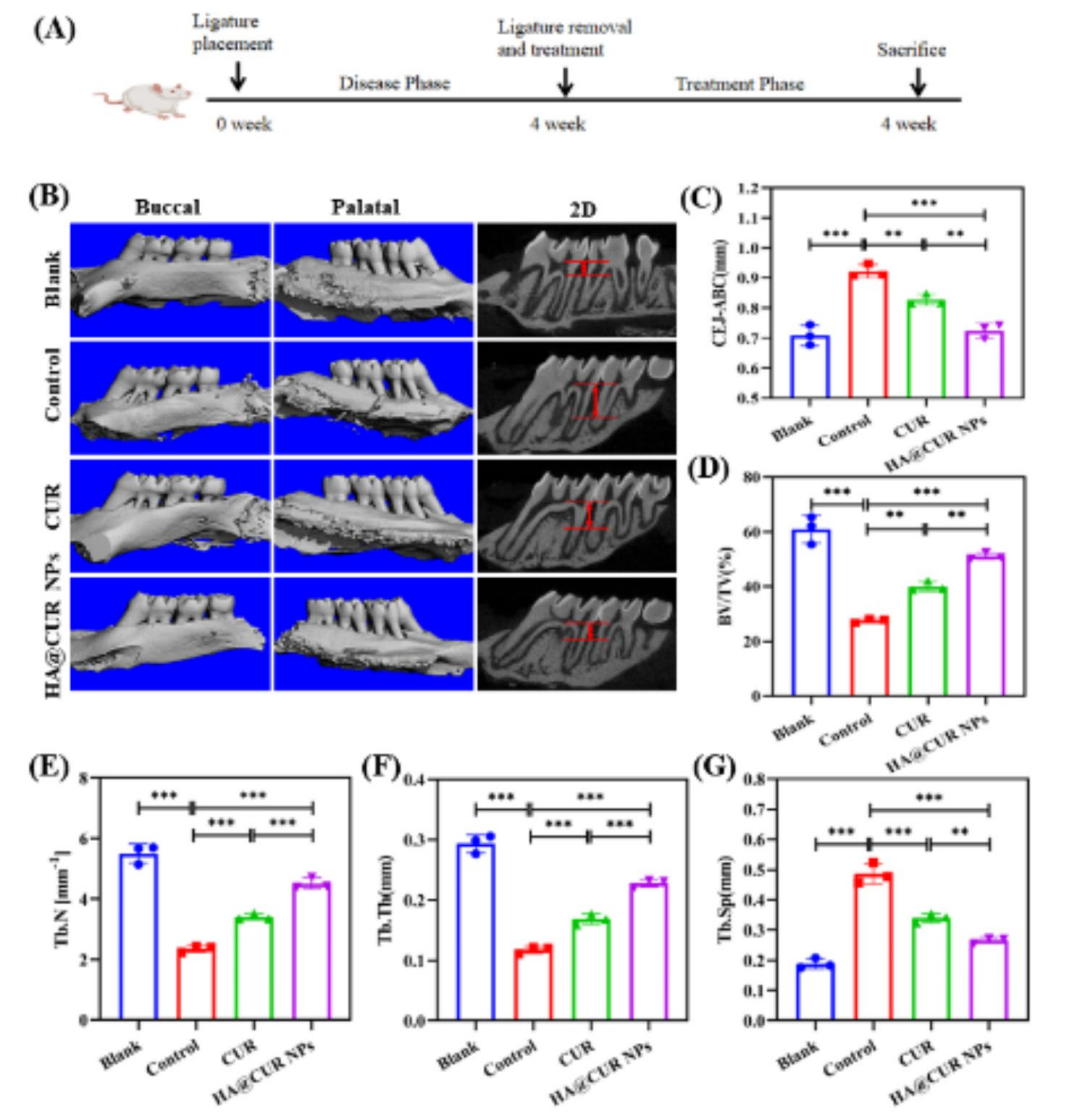
(**A**) Schematic of the rat periodontitis treatment process. (**B**) Three-dimensional reconstructed images and two-dimensional sagittal plane X-ray images of maxillary alveolar bone on the buccal and palatal sides under different experimental conditions; scale bar: 1.0 mm. Mean values of bone quality parameters for each treatment group: (**C**) CEJ-ABC, (**D**) BV/TV, (**E**) Tb.N, (**F**) Tb.Th, and (**G**) Tb.Sp. (*n* = 3) (*n.s.*: not significant; *p < 0.05; **p < 0.01; ***p < 0.001)

Given the positive effects of HA@CUR NPs on the treatment of periodontitis, we constructed a rat ligature model to evaluate the in vivo therapeutic efficacy of HA@CUR NPs (Fig. 7A). Bilateral maxillary first molars of Sprague-Dawley (SD) rats were ligatured to establish the periodontitis model [34]. The 3D reconstruction images and sagittal views are displayed in Fig. 7A. We observed gradual resorption of the alveolar bone around the maxillary first molar after ligation. Furthermore, we measured and analyzed the distance between the cemento-enamel junction (CEJ) and the alveolar bone crest (ABC) at six points for each group. The distance of CEJ-ABC significantly increased in the control group, indicating the successful establishment of the periodontitis model. The long-lasting retention function of HA-based carriers for periodontal disease tissues was investigated prior to performing the treatment. From the results (Figure S10), 24 h after local injection, the fluorescence intensity of the HA-Cy5.5 NPs group was twice higher than that of the free Cy5.5 group. This was attributed to the strong interaction force between HA and CD44 expressed by the tissue cells (discussed in the previous section). After treatment with free CUR, the CEJ-ABC distance was reduced but not as significantly as in the HA@CUR NPs treated group. These results suggest that HA@CUR NPs can improve bone loss induced by ligature-induced periodontitis (Fig. 7B). On the other hand, the mean values of CEJ-ABC, trabecular separation (Tb.Sp), trabecular number (Tb.N), trabecular thickness (Tb.Th), and bone volume/tissue volume (BV/TV) for various treatment groups were be counted to quantify the therapeutic effects. The analysis revealed (Fig. 7C and G) that the HA@CUR NPs group showed more significant improvements in metrics including CEJ-ABC, Tb.Sp, Tb.N, Tb.Th, and BV/TV, compared to free CUR group. These outcomes can be attributed to enhanced uptake of HA@CUR NPs at lesion sites and increased bioavailability resulting from improved CUR solubility. HA@CUR NPs not only effectively ameliorated the inflammatory environment but also possessed the ability to clear excessive ROS at the lesion site. Consequently, HA@CUR NPs exhibit potent efficacy in improving the inflammatory microenvironment and promoting regeneration of alveolar bone.

**Fig. 8:**
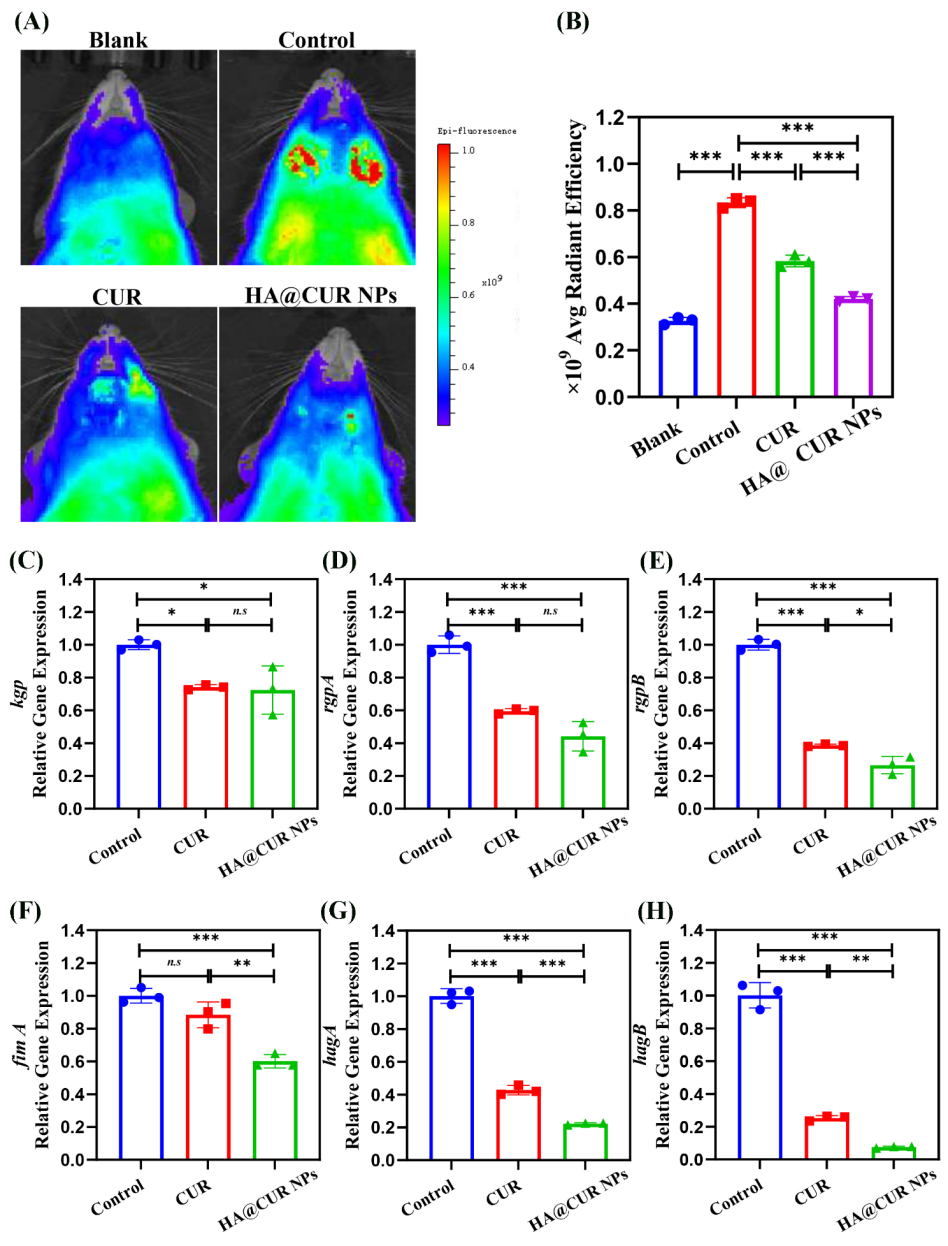
(**A**, **B**) The ability of HA@CUR NPs to scavenge ROS was observed by fluorescence imaging using an in vivo imaging system as a test device, which then yielded relative semi-quantitative information. (**C-H**) RNA expression levels of bacterial virulence factors in periodontal tissues (*n* = 3). (*n.s.*: not significant; *p < 0.05; **p < 0.01; ***p < 0.001)

To validate the ROS scavenging ability of HA@CUR NPs in vivo, a live imaging system was used to monitor ROS levels at the ligation site in reference to a previous report for in vivo NPs scavenging efficacy assessment [35]. Observation of the in vivo image system showed that strong fluorescent signals were detected around the DCFH-DA injection site in the positive control group (Fig. 8A). Notably, the group treated with both HA@CUR NPs and free CUR exhibited lower fluorescence intensity and smaller areas of dense signal compared with the positive control group. In addition, a graphical representation of the statistical results based on the average radiation efficiency of the ROS signals showed that the ROS clearance efficiency of the HA@CUR NPs was slightly better than the reduction rate of the free CUR group (Fig. 8B). Both formulations were able to effectively remove most of the ROS generated in the tissues, indicating that HA@CUR NPs have excellent ROS removal ability at the periodontitis site.

On the another hand, periodontitis is caused by the colonization of the oral cavity by selective Gram-negative bacteria, particularly *P.g* [36]. *P.g.* possesses a variety of virulence factors that contribute to the invasion of host tissues, thereby deregulating the host immune system [37]. CUR inhibits periodontal bacteria and *P.g.* virulence factors in a dose-dependent manner [38]. Therefore, here we investigated the in vivo antimicrobial activity of HA@CUR NPs by studying the effect of HA@CUR NPs on the RNA expression levels of *P.g*. virulence factor. The results showed that HA@CUR NPs were able to significantly reduce the RNA expression of various virulence factors in *P.g.* And the inhibition efficiency of HA@CUR NPs was higher than that of free CUR group in *rgpB*, *fimA*, *hagA*, and *hagB* (Fig. 8C and H). These results shown that HA@CUR NPs could scavenge ROS as well as inhibit the expression of *P.g.* virulence factors in the focal tissues, thereby promoting healing of periodontitis.

### Histological analysis

**Fig. 9:**
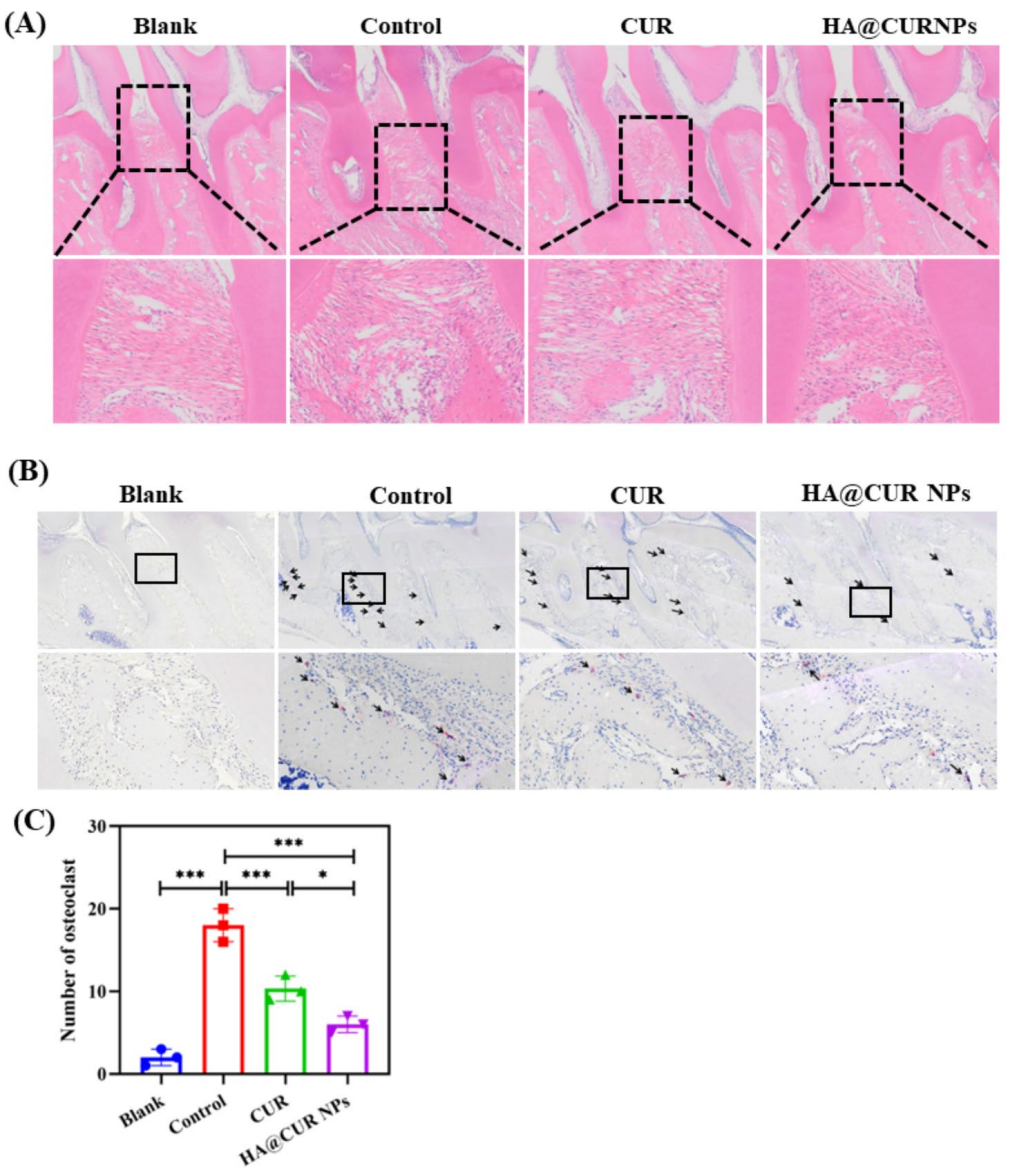
(**A**) H&E and (**B**) TRAP staining in the interdental area of the maxillary first and second molars.Note: Black arrows in the images indicate osteoclasts (cells positive for TRAP staining with three or more nuclei). (**C**) Quantitative measurements of osteoclast numbers on the alveolar bone surface in the region of the maxillary first and second molars. (*n* = 3) (*n.s.*: not significant; *p < 0.05; **p < 0.01; ***p < 0.001)

To further confirm the results mentioned above, we performed H&E and TRAP staining to assess the inflammatory status and periodontal regeneration. H&E staining images (Fig. 9A) showed regular thickness of the epithelial layer, dense connective tissue, well-arranged collagen fibres, and smooth alveolar bone surface in the periodontal tissues of blank group (un-ligated) animals. In contrast, the control group had increased epithelial layer thickness, structural disorganisation, marked cellular infiltration, severe destruction of the alveolar ridge apex, and irregular alveolar bone surface. Compared with the control group, HA@CUR NPs group and CUR group showed reduced inflammatory cell infiltration, lesser tissue destruction, decreased alveolar bone resorption, significantly attenuated periodontitis, and slowed progression of inflammation.

Pathological bone resorption is related to an imbalance between osteoclast-mediated bone destruction and osteoblast-mediated bone formation, and regulating osteoclast activity is crucial for treating bone resorptive diseases [39]. Here, we performed TRAP staining and quantified the number of osteoclasts to study the anti-osteoclastic effects of free CUR and HA@CUR NPs (as shown in Fig. 9B and C). In the control group, we observed a large number of TRAP-positive cells (Fig. 9B, black arrows) forming resorption fissures along the alveolar bone surface around the maxillary first and second molars. After magnification, osteoclasts with more than three nuclei could be seen. This suggests that periodontitis leads to the presence of large numbers of osteoclasts. In the normal group, only a few TRAP-positive cells were present (Fig. 9) with numbers being (2.0 ± 1.0). Compared to the control group, treatment with free CUR effectively reduced the number of TRAP-positive cells (10.3 ± 1.5). However, the number of TRAP-positive cells in the CUR group (6.0 ± 1.0) was significantly higher than HA@CUR NPs group. This indicates that HA@CUR NPs can effectively inhibit the production of osteoclasts and promote recovery at the periodontitis lesion sites.

**Fig. 10:**
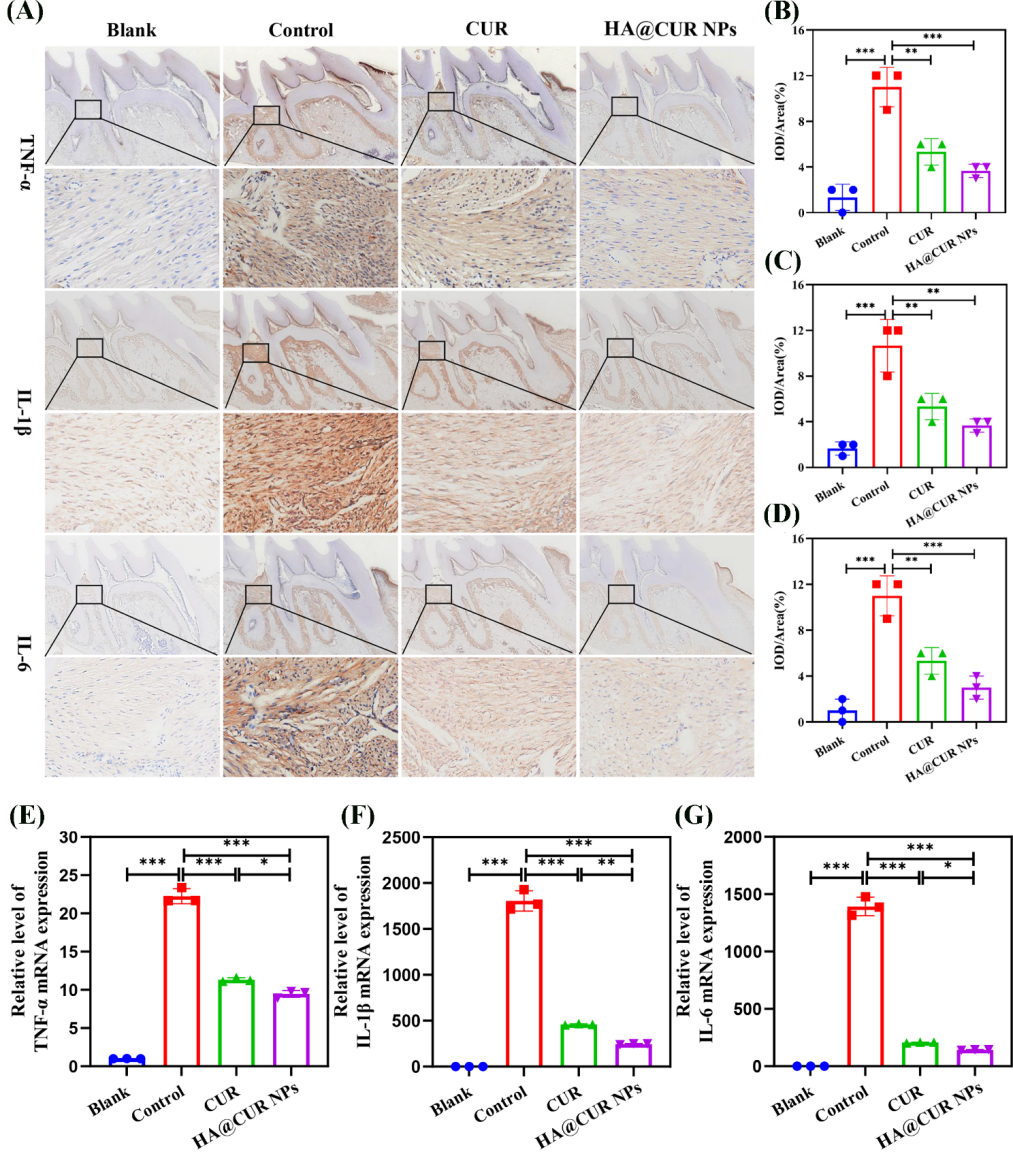
(**A**) Representative immunohistochemical staining images of TNF-α, IL-1β, and IL-6 in the maxillary bone after treatment. Quantitative analysis of staining intensity for (**B**) TNF-α, (**C**) IL-1β, and (**D**) IL-6. RNA expression levels of (**E**) TNF-α, (**F**) IL-1β, and (**G**) IL-6 in periodontal tissues (*n* = 3). (*n.s.*: not significant; *p < 0.05; **p < 0.01; ***p < 0.001)

It is well-known that the inflammatory environment caused by is a significant factor leading to bone resorption [40]. CUR, as a natural product with anti-inflammatory properties, can effectively suppress inflammation at periodontal sites. To confirm that CUR released from NPs can inhibit periodontal inflammation, the response to inflammation was observed by assessing the expression levels of TNF-α, IL-1β, and IL-6 in IHC-stained sections (Fig. 10A). Treatment with free CUR and HA@CUR NPs significantly reduced the expression levels of TNF-α, IL-1β, and IL-6 in periodontal tissues. Quantification of the levels of each inflammatory factor (Fig. 10B and D) revealed that both free CUR and HA@CUR NPs effectively decreased the severity of inflammation. These results suggest that during treatment, HA@CUR NPs can effectively maintain anti-inflammatory effects, providing favorable conditions for periodontal regeneration. To further validate the anti-inflammatory effects of HA@CUR NPs, TNF-α, IL-1β, and IL-6 RNA expression levels in periodontal tissues were investigated. From the results (Fig. 10E and G), free CUR and HA@CUR NPs were able to effectively inhibit the expression of inflammatory factors. These results confirmed the in vivo anti-inflammatory activity of HA@CUR NPs.

**Fig. 11:**
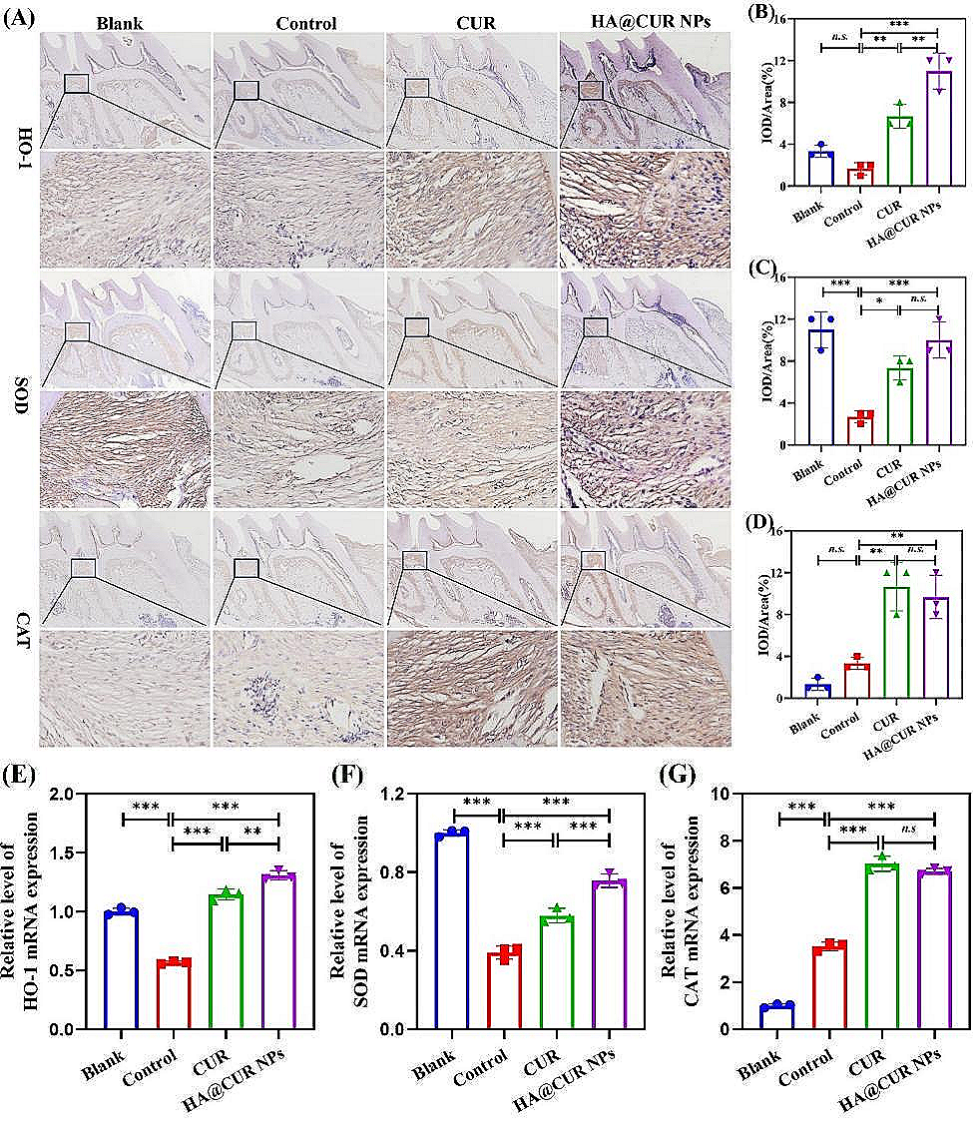
Representative immunohistochemical staining images of HO-1, SOD, and CAT in the maxilla following treatment (**A**) Quantitative analysis of staining intensity for (**B**) HO-1, (**C**) SOD, and (**D**) CAT. RNA expression levels of (**E**) HO-1, (**F**) SOD, and (**G**) CAT in periodontal tissues (*n* = 3). (*n.s.*: not significant; *p < 0.05; **p < 0.01; ***p < 0.001)

On the other hand, oxidative stress, as one of the key characteristics of periodontitis, has also garnered significant attention for its impact on periodontal tissues. The study included immunohistochemical (IHC) analysis of the expression levels of HO-1, SOD, and CAT (Fig. 11A). The results indicated a marked increase in the expression levels of HO-1, SOD, and CAT in the area between the first and second molars in both the free CUR and HA@CUR NPs treatment groups. By quantifying these three metrics (Fig. 11B and D), it was also clearly found that the expression levels of antioxidant factors HO-1, SOD, and CAT were significantly improved in the free CUR and HA@CUR NPs-treated groups compared with the control group. RNA levels of HO-1, SOD, and CAT were significantly increased in periodontal tissue after treatment with HA@CUR NPs. This result is consistent with the findings obtained by IHC (Fig. 11E and G). These suggests that HA@CUR NPs not only provide effective alleviation of inflammation at the affected sites but also diminish oxidative stress at the lesion locations, thereby further ensuring recovery from periodontal inflammation.

### In vivo biocompatibility assessment of HA@CUR NPs

The long-term toxicity of HA@CUR NPs was evaluated after local administration in the periodontal pocket. Observations indicated no significant change in the body weight of the tested rats during the treatment period (Figure S14). H&E staining of the heart, liver, spleen, lungs, and kidneys demonstrated that topical administration of HA@CUR NPs did not cause damage to major organs, e.g., morphological changes (Figure S11). Myocardial fibers were arranged in a ladder pattern without signs of inflammatory cell infiltration. Hepatocytes were orderly without any histopathological changes observed. The splenic red and white pulp structure was distinct with no anomalies. Alveolar structures were clear with no evidence of edema or inflammatory cell infiltration. Glomeruli were uniform in size with a normal morphology and clear boundaries, indicating a normal renal physiological structure. Furthermore, results from liver and kidney function tests and routine blood parameters (Figure S12, S13) were within the normal range, with no statistical differences observed between groups. Hence, HA@CUR NPs not only effectively treat inflammation at periodontal sites and improve the oxidative stress condition at lesions but also constitute a nanomedicine with good biocompatibility.

## Conclusion

In summary, we have successfully developed a nanocarrier with excellent biocompatibility and ROS responsiveness. The HA@CUR NPs enhance cellular uptake at inflamed sites and sustain drug release at the lesion, effectively inhibiting the progression of periodontitis. The release of CUR from the core of HA@CUR NPs achieves multiple biological functions, such as anti-microbial activity, alleviation of inflammation, scavenging of excess ROS, and immunomodulation. Additionally, HA@CUR NPs demonstrate good biocompatibility with no significant side effects in vitro and in vivo. Notably, the HA used in the NPs can achieve long-lasting retention in the oral cavity. This not only guarantees the treatment of periodontitis, but also brings favorable factors to the patient’s compliance with the long oral retention. Therefore, HA@CUR NPs as a novel multifunctional nanocarrier (ROS scavenging, inflammation modulation, antimicrobial, and long-lasting retention) provides a reference for the clinical application in the treatment of periodontitis.

### Electronic supplementary material

Below is the link to the electronic supplementary material.

Supplementary Material 1

## Data Availability

All data contained in the study are in this article.
