# Supplementary material for: The application of phenylboronic acid pinacol ester functionalized ROS-responsive multifunctional nanoparticles in the treatment of Periodontitis
Source: J Nanobiotechnology. 2024 Apr 15;22:181. doi: 10.1186/s12951-024-02461-0 (PMC11017612; doi:10.1186/s12951-024-02461-0)
Supplement: Supplementary file 1 — Supplementary Material 1 [file 12951_2024_2461_MOESM1_ESM.docx]

**Supporting Information for**

**The Application of Phenylboronic Acid Pinacol Ester Functionalized ROS-Responsive Multifunctional Nanoparticles in the Treatment of Periodontitis**

Jinhong Chen^a, #^, Aihua Luo ^a, #^, Mengmeng Xu^a^, Yao Zhang^a^, Zheng Wang^a^, Shuang Yu^a^, Li Zhu^b,^*, Wei Wu^b,^*, Deqin Yang^a,^*

*^a^ Department of Endodontics, Stomatological Hospital of Chongqing Medical University, Chongqing 404100, China,*

*^b^ Key Laboratory of Biorheological Science and Technology, Ministry of Education, College of Bioengineering, Chongqing University, Chongqing, 400044,China.*

# These authors contributed equally to this work.

***Corresponding authors:**

***Email*:** [yangdeqin@hospital.cqmu.edu.cn](mailto:yangdeqin@hospital.cqmu.edu.cn) **(Deqin Yang)**; david2015@cqu.edu.cn (**Wei Wu**); zhuli0109@126.com (**Li Zhu**)

**This file includes:**

**Figures S1~S13**

**Tables S1~S3**


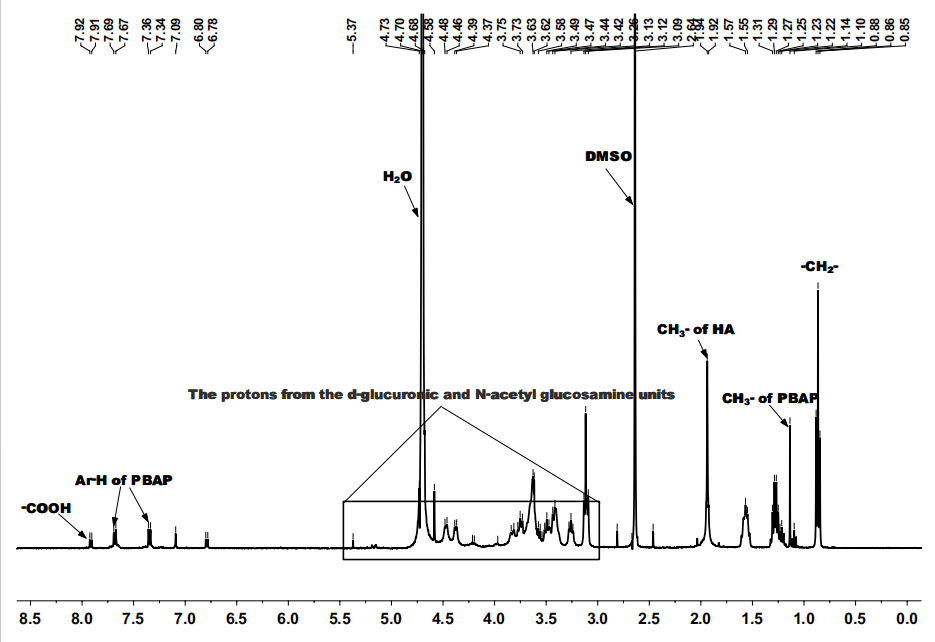


**Figure S1.** ^1^H NMR spectrum of PBAP-CDI-HA.





**Figure S2.** FT-IR spectra of HA, PBAP, and HA-CDI-PBAP.


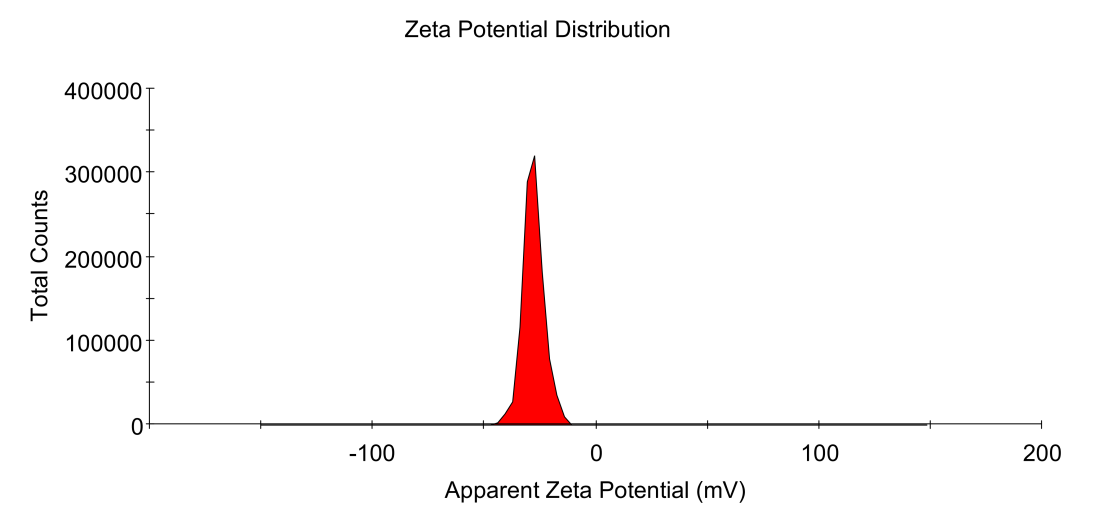


**Figure S3.** Zeta potential of HA@CUR NPs.





**Figure S4.** FT-IR spectra of HA@CUR NPs, Blank NPs and CUR.





**Figure S5.** XRD patterns of HA@CUR NPs, Blank NPs, and CUR.


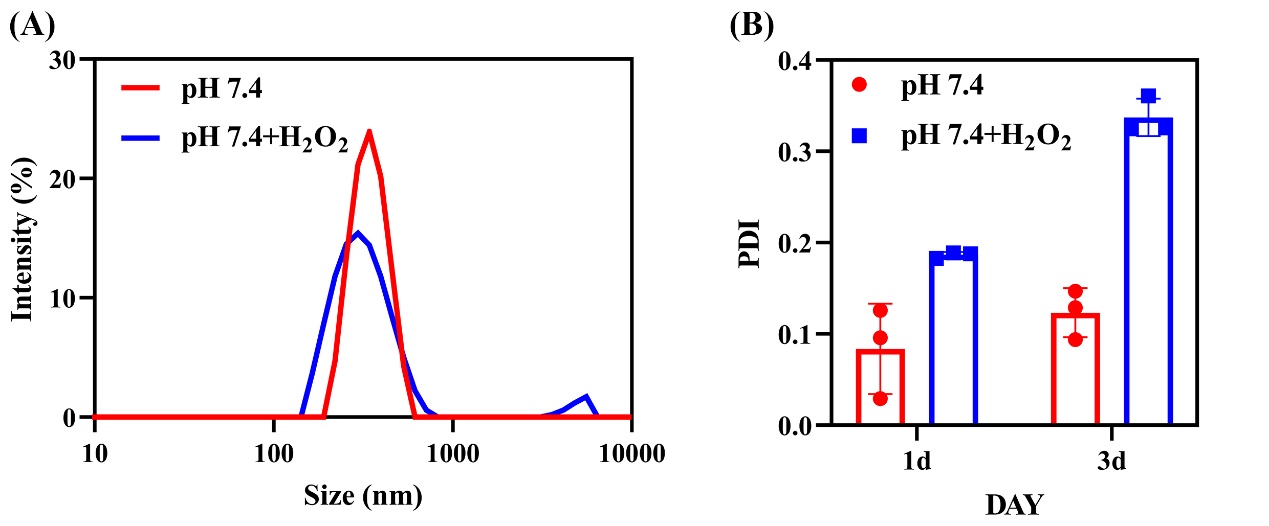


**Figure S6.**(A) Particle size distribution of HA@CUR NPs in pH 7.4 and pH 7.4+H_2_O_2_. (B) PDI of HA@CUR NPs at pH 7.4 and pH 7.4+H_2_O_2_ on days 1 and 3.


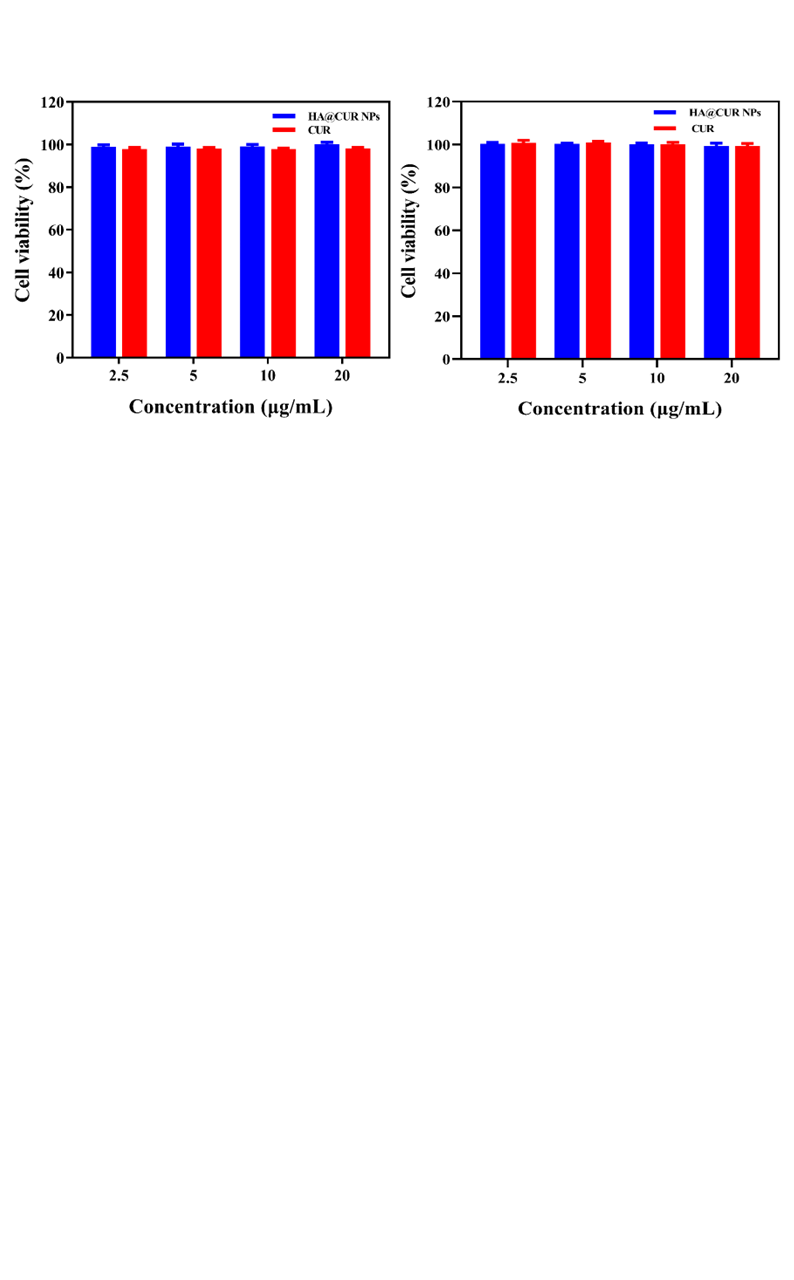


**Figure S7.** Survival of (A) RAW 264.7 macrophages and (B) HGF fibroblasts when RAW 264.7 cells and HGF cells were treated with different concentrations of HA@CUR NPs and CUR.


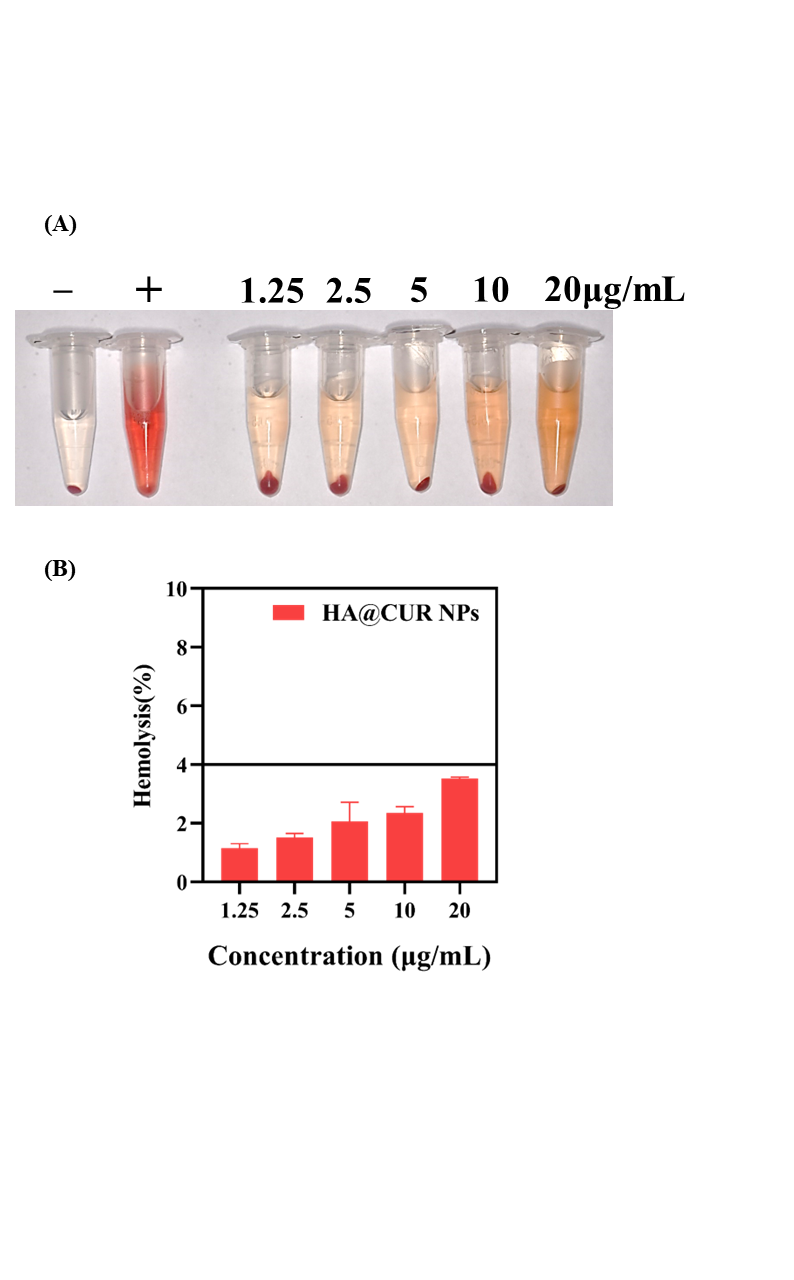


**Figure S8.** (A) Hemolysis images and (B) hemolysis rate of HA@CUR NPs.


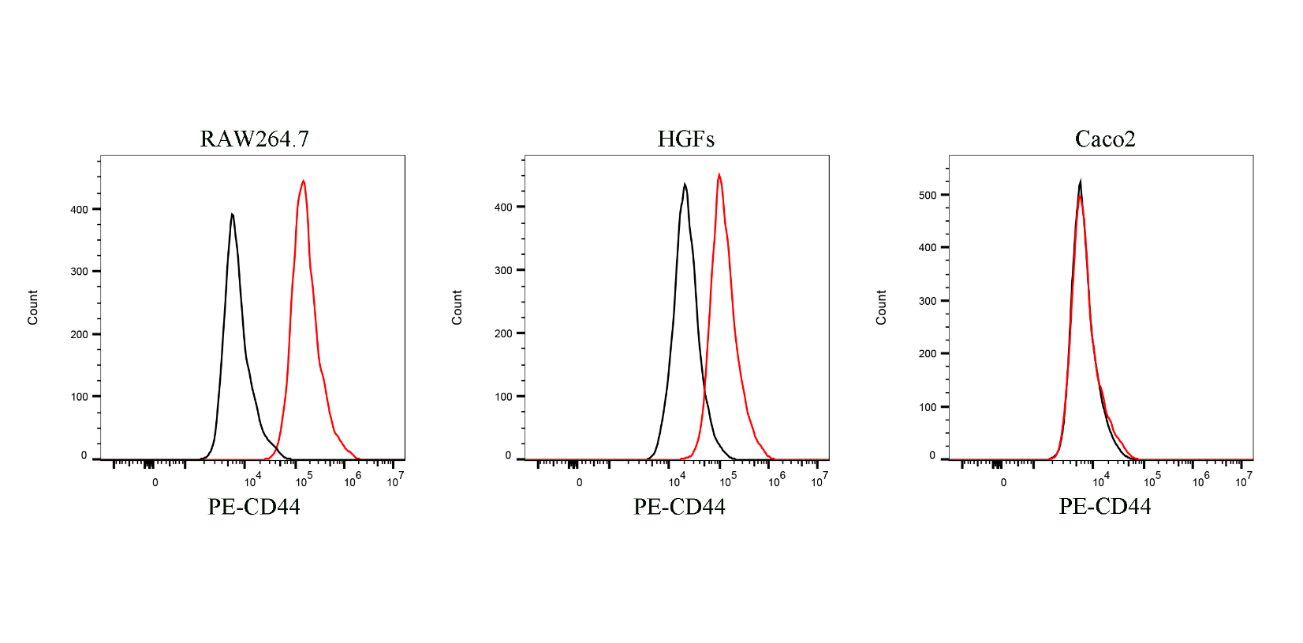


**Figure S9.** Flow cytometry analysis of the expression levels of CD44 in RAW 264.7, HGFs and Caco2 cell


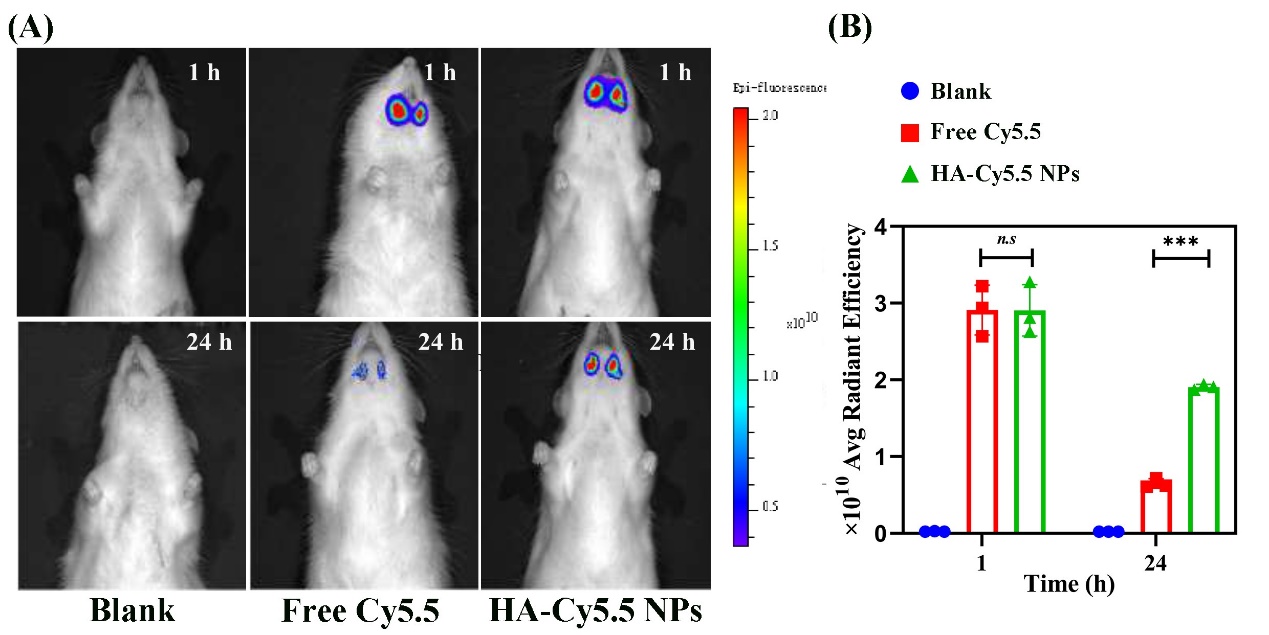


**Figure S10.** (A) *In vivo* fluorescence images and (B) fluorescence semi-quantitative values of blank, free Cy5.5, and HA-Cy5.5 NPs at different times


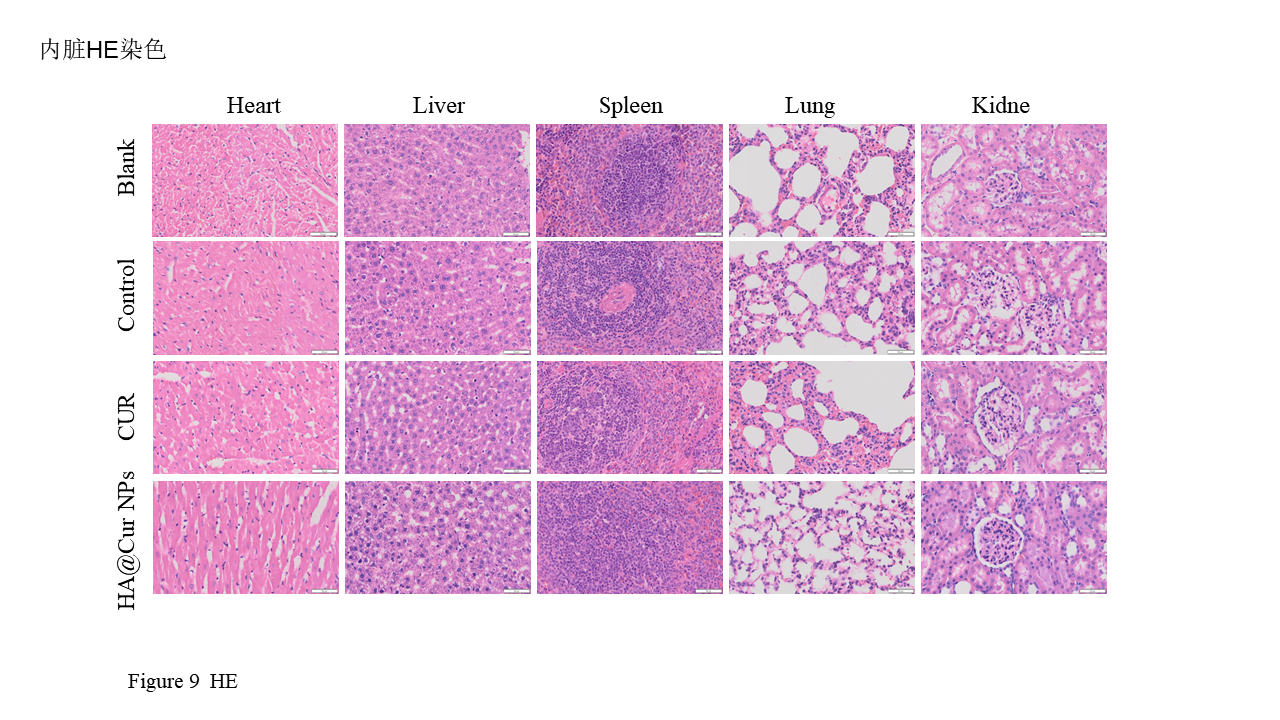


**Figure S11.** *In vivo* biocompatibility assessment of HA@CUR NPs and CUR. HE staining of major organs (heart, liver, spleen, lung, kidney) in different groups of animals.

**
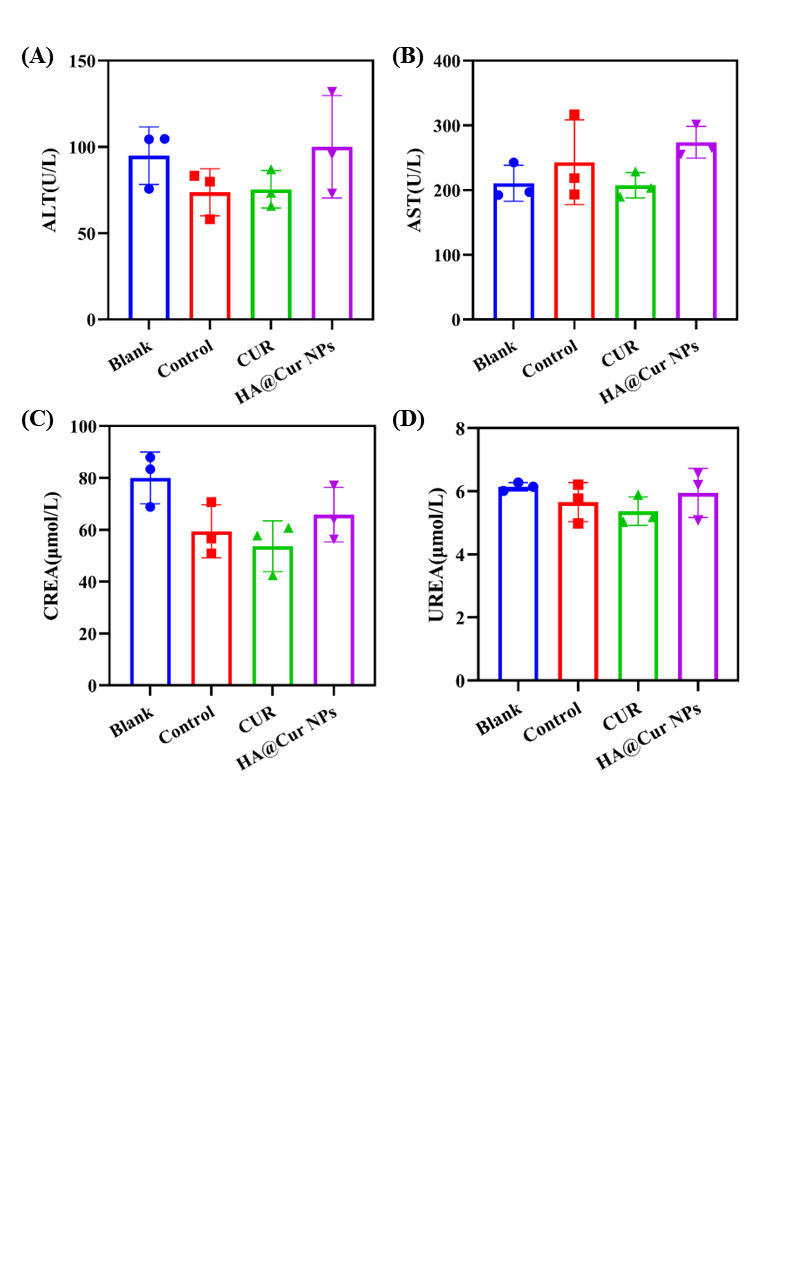
**

**Figure S12.** *In vivo* biocompatibility assessment of HA@CUR NPs and CUR. Statistical analysis of liver and kidney function indicators (ALT, AST, CREA, UREA) in animals.


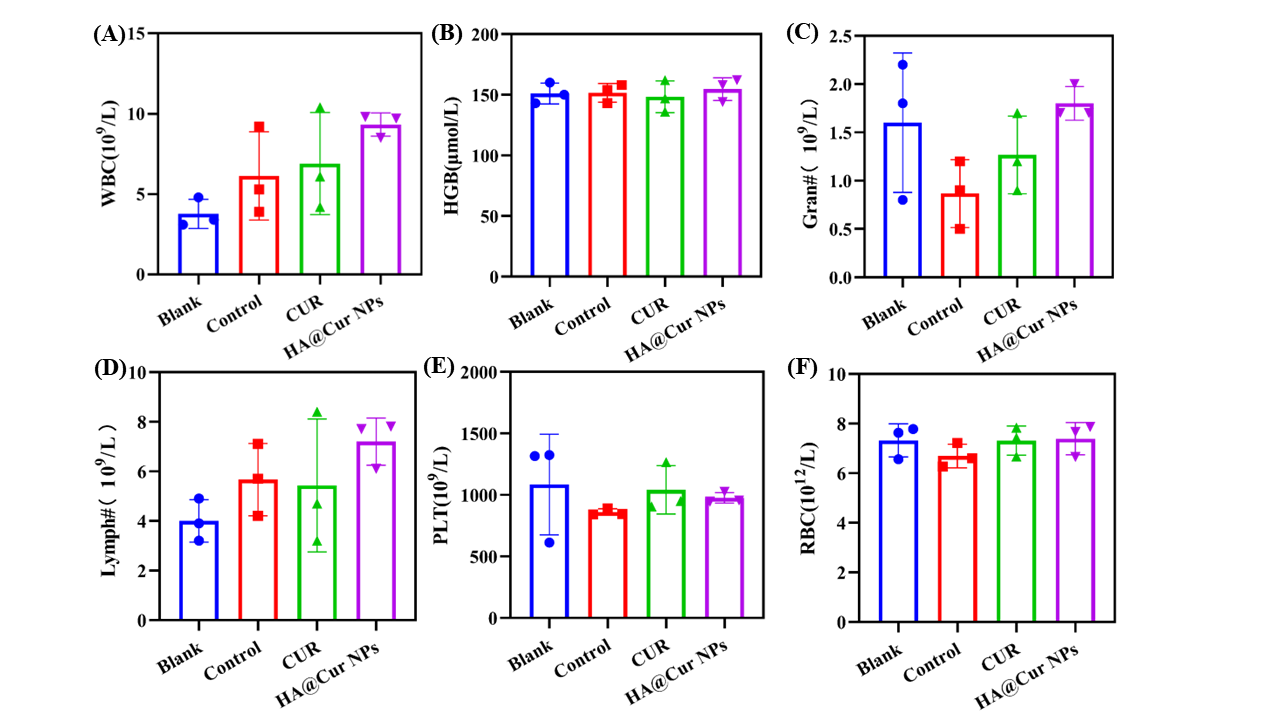


**Figure S13.** *In vivo* biocompatibility assessment of HA@CUR NPs and CUR. Statistical analysis of blood routine indicators (WBC, HGB, Lymph#, Gran#, RBC, PLT) in animals.

**
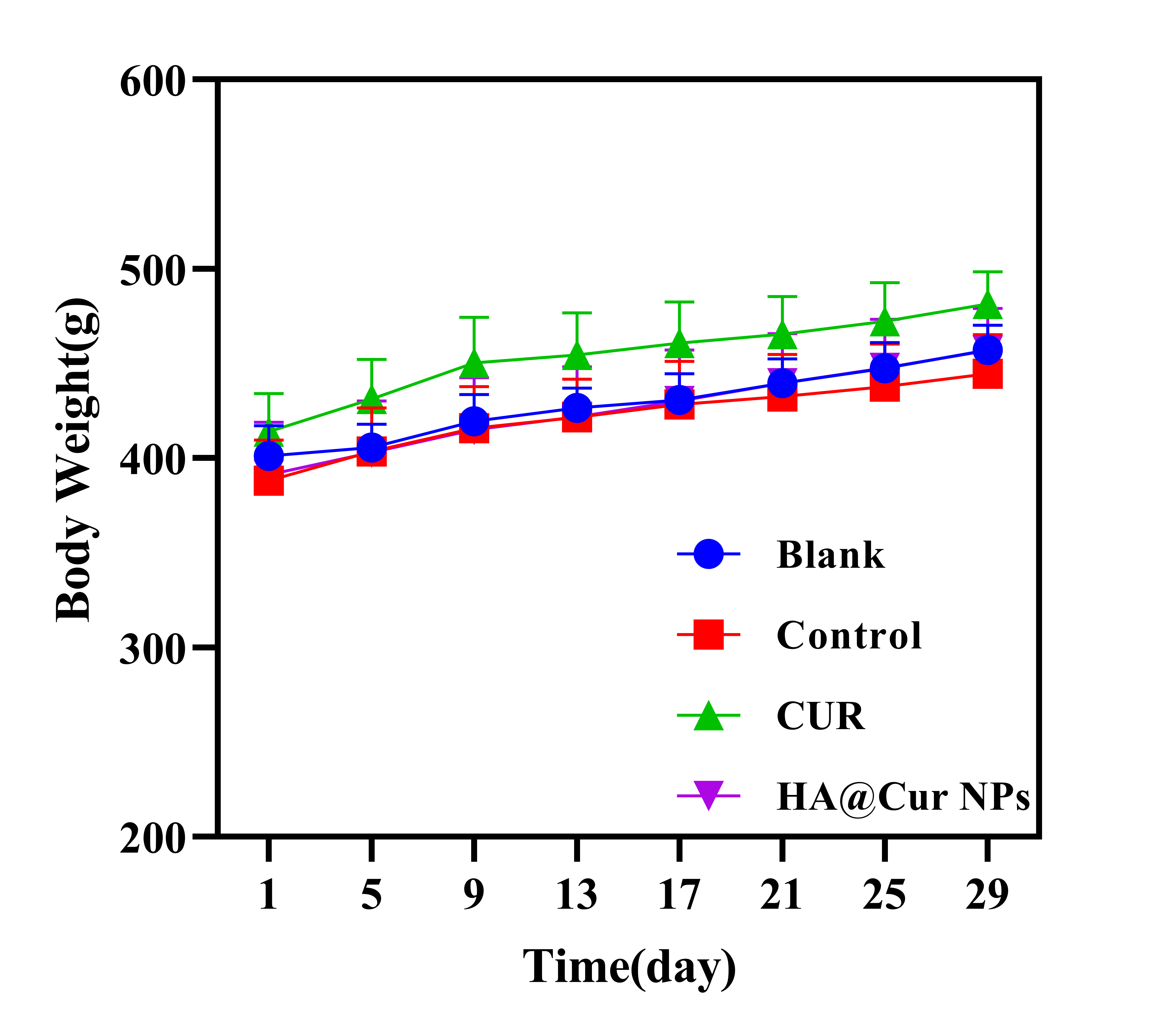
**

**Figure S14.** *In vivo* biocompatibility assessment of HA@CUR NPs and CUR. Statistical analysis of body weight changes in animals during drug injection.

**Table S1. Primer sequences were applied in this study.**

| **Gene** | **sequences** |
| --- | --- |
| GADPH | 5’-GACATCAAGAAGGTGGTGAAGC-3’  5’-GAAGGTGGAAGAGTGGGAGTT-3’ |
| TNF-α | 5’-CACCACGCTCTTCTGTCTACTG-3’  5’-GGTCTGGGCCATAGAACTGA-3’ |
| IL-1β | 5’-TTGAAGTTGACGGACCCCA-3’  5’-GAGTGATACTGCCTGCCTGAAG-3’ |
| IL-6 | 5’-GTTGCCTTCTTGGGACTGATG-3’  5’-TTGGGAGTGGTATCCTCTGTGA-3’ |
| Arg1 | 5’-GCATATCTGCCAAAGACATCGT-3’  5’-CCATCACCTTGCCAATCCC-3’ |
| iNOS | 5’-TGGAGCGAGTTGTGGATTGT-3’  5’-TCTCTGCCTATCCGTCTCGTC-3’ |
| COX-2 | 5’-GGTCATTGGTGGAGAGGTGTATC-3’  5’-TGCTCCTGCTTGAGTATGTCG-3’ |
| Mmp8 | 5’-GACTGTCTCCACATTTTGCCG-3’  5’-ACAGGGAGAAGCAGACATCAAC-3’ |
| HO-1 | 5’-TTACCTTCCCGAACATCGAC-3’  5’-TCCTCTGTCAGCATCACCTG-3’ |
| SOD | 5’-CTGGAGCCACACATTAACGC-3’  5’-CGGTGGCGTTGAGATTGTTC-3’ |
| CAT | 5’-AGAGGAAACGCCTGTGTGAG-3’  5’-GCGTGTAGGTGTGAATTGCG-3’ |

**Table S2. Primer sequences were applied in this study (rat).**

| **Gene** | **sequences** |
| --- | --- |
| GADPH | 5’ -ACAGTCCATGCCATCACTGCC -3’  5’-GCCTGCTTCACCACCTTCTTG-3’ |
| TNF-α | 5’-TCC CAA CAA GGA GGA GAA GT-3’  5’-TGG TAT GAA GTG GCA AAT CG-3’ |
| IL-1β | 5’-AACCTGCTGGTGTGTGACGTTC-3’  5’-CAGCACGAGGCTTTTTTGTTGT-3’ |
| IL-6 | 5’- GTCTTCTGGAGTTCCGTTTCT -3’  5’- GCCGAGTAGACCTCATAGTGA -3’ |
| CAT | 5’-GCATATCTGCCAAAGACATCGT-3’  5’-CCATCACCTTGCCAATCCC-3’ |
| SOD | 5’-TGGAGCGAGTTGTGGATTGT-3’  5’-TCTCTGCCTATCCGTCTCGTC-3’ |
| HO-1 | 5’-GGTCATTGGTGGAGAGGTGTATC-3’  5’-TGCTCCTGCTTGAGTATGTCG-3’ |

**Table S3. Primer sequences were applied in this study (Bacteria)**

| **Gene** | **sequences** |
| --- | --- |
| 16S rRNA | 5′-TGTAGATGACTGATGGTGAAA-3′ 5′-ACTGTTAGCAACTACCGATGT-3′ |
| fimA | 5′-CAGCAGGAAGCCATCAAATC-3′  5′-CAGTCAGTTCAGTTGTCAAT-3′ |
| hagA | 5′-ACAGCATCAGCCGATATTCC-3′  5′ -CGAATTCATTGCCACCTTCT-3′ |
| hagB | 5′-TGTCGCACGGCAAATATCGCTAAAC-3′  5′-CTGGCTGTCCTCGTCGAAAGCATAC-3′ |
| kgp | 5′-AGCTGACAAAGGTGGAGACCAAAGG-3′  5′-TGTGGCATGAGTTTTTCGGAACCGT-3′ |
| rgpA | 5′-GCCGAGATTGTTCTTGAAGC-3′  5′ -AGGAGCAGCAATTGCAAAG-3′ |
| rgpB | 5′-CGCTGATGAAACGAACTTGA-3′  5′ -CTTCGAATACCATGCGGTT-3′ |
